# A Rice Gene of *De Novo* Origin Negatively Regulates Pathogen-Induced Defense Response

**DOI:** 10.1371/journal.pone.0004603

**Published:** 2009-02-25

**Authors:** Wenfei Xiao, Hongbo Liu, Yu Li, Xianghua Li, Caiguo Xu, Manyuan Long, Shiping Wang

**Affiliations:** 1 National Key Laboratory of Crop Genetic Improvement, National Center of Plant Gene Research (Wuhan), Huazhong Agricultural University, Wuhan, China; 2 Department of Ecology and Evolution, University of Chicago, Chicago, Illinois, United States of America; Cairo University, Egypt

## Abstract

How defense genes originated with the evolution of their specific pathogen-responsive traits remains an important problem. It is generally known that a form of duplication can generate new genes, suggesting that a new gene usually evolves from an ancestral gene. However, we show that a new defense gene in plants may evolve by *de novo* origination, resulting in sophisticated disease-resistant functions in rice. Analyses of gene evolution showed that this new gene, *OsDR10*, had homologs only in the closest relative, *Leersia* genus, but not other subfamilies of the grass family; therefore, it is a rice tribe-specific gene that may have originated *de novo* in the tribe. We further show that this gene may evolve a highly conservative rice-specific function that contributes to the regulation difference between rice and other plant species in response to pathogen infections. Biologic analyses including gene silencing, pathologic analysis, and mutant characterization by transformation showed that the *OsDR10*-suppressed plants enhanced resistance to a broad spectrum of *Xanthomonas oryzae* pv. *oryzae* strains, which cause bacterial blight disease. This enhanced disease resistance was accompanied by increased accumulation of endogenous salicylic acid (SA) and suppressed accumulation of endogenous jasmonic acid (JA) as well as modified expression of a subset of defense-responsive genes functioning both upstream and downstream of SA and JA. These data and analyses provide fresh insights into the new biologic and evolutionary processes of a *de novo* gene recruited rapidly.

## Introduction

To resist pathogen attacks, plants have evolved an efficient defense response system to elude or minimize the effects of diseases. Many genes are involved in this defense system. The major disease resistance (*R*) genes can directly or indirectly recognize the corresponding pathogen avirulence factors and trigger a highly specific resistance [1]. The defense-responsive or defense-related genes also play an important role in the regulation of plant defense responses. Most of them encode the components of defense signal transduction pathways and provide nonspecific resistance to a broad spectrum of pathogens [2], [3]. An increasing number of *R* genes and defense-responsive genes encoding similar proteins have been identified as involved in plant–pathogen interactions in both dicots and monocots. The accumulated evidence, however, suggests that the defense responses to pathogen infection are not necessarily the same in dicots and monocots. For example, a rise in the level of endogenous salicylic acid (SA) is required for systemic acquired resistance in dicots, but rice (*Oryza sativa* L.), a model monocot, maintains a high level of SA without activating defense responses [4]. Activation of indole-3-acetic acid-amido synthetase can enhance disease resistance in both *Arabidopsis* (a model dicot) and rice, but the former is associated with an increase in SA and the latter is SA independent [5], [6]. Identification of species-specific genes will help to elucidate the differences in pathogen-induced defense responses among plant species.

A species-specific gene is the one that recently evolved or in rare cases the one that originated in the remote past but was lost in all other related species. A new *R* gene evolved a new and species-specific disease resistance trait. A number of mechanisms that can generate new genes are known [7]. These mechanisms are often dependent on the duplication of ancestral genes or ancestral genomic sequences. Thus, the genes that originated from these mechanisms would have additional homologous gene copies in the same species or different species. However, recent studies in *Drosophila melanogaster*, *D. simulans*, *D. yakuba*, *D. erecta*, and *Saccharomyces cerevisiae* detected certain genes that have no homologs even in the species that diverged very recently [8]–[12]. This raised the possibility of a *de novo* origination of new genes, to suggest the possibility that they may derive from ancestrally noncoding sequences or other unknown mechanisms [8], [10], although there is much to do in understanding the biology of these *de novo* genes. It was speculated that many so-called “orphan” genes might actually be *de novo* genes [13]; http://blogs.nature.com/nature/journalclub/2007/10.

The *de novo* evolution of genes is considered an important process in the genomes of some prokaryotes. Approximately 12% of genes in some archeal and proteobacterial species are thought to have *de novo* origination based on a computational analysis [14]; however, the origin of these *de novo* genes is unknown. Levine et al. [8] first reported *de novo* origination of whole genes from expressed noncoding regions in *D. melanogaster* and *D. simulans*. Other *de novo* genes from noncoding regions were then discovered in *D. yakuba* and *D. erecta* [9], [10] as well as in *S. cerevisiae* [12]. In comparison, the *hydra* gene in *D. melanogaster* was suggested to have evolved *de novo* from a DNA sequence that inserted into its present site; repetitive sequences in this region may have contributed to structural and expression-level evolution of this gene [11]. Subsequently, we asked if plants have *de novo* genes. If they do, we can use the well-developed biologic analyses to further investigate their biologic processes to reveal how a function can be generated by a *de novo* gene. The defense system in rice is an excellent system to test this possibility because of the availability of rich analysis tools and knowledge about the disease resistance within the rice species.

Bacterial blight of rice, caused by *Xanthomonas oryzae* pv. *oryzae* (*Xoo*), is one of the most serious diseases of rice worldwide. Rice resistance to *Xoo* is governed by both *R* genes and resistance quantitative trait loci (QTLs). To date, six *R* genes for bacterial blight resistance have been isolated and numerous QTLs for bacterial blight resistance have been identified [15]–[17]. However, only a few genes underlying the resistance QTLs are characterized [18]. Our previous study showed that the cDNA clone BI71N2 (a fragment of *Os08g05960*, according to the rice genome annotation of The Institute for Genomic Research [TIGR], http://rice.tigr.org) showed suppressed expression in response to pathogen infection in different resistance-cultivated rice lines carrying an *R* gene for resistance to *Xoo* or *Magnaporthe grisea*, which causes fungal blast, another devastating rice disease worldwide [3]. In addition, BI71N2 colocalizes with a previously identified resistance QTL in rice chromosome 8 [17]. Those studies suggested that the gene represented by BI71N2 may be a negative regulator of disease resistance in a pathogen-nonspecific way, but the activation of *R* genes is the key to suppressing its expression. This finding also implies that suppression of the gene represented by BI71N2 in susceptible rice without the existence of an *R* gene may increase rice resistance to pathogen infections.

To test the above hypothesis, we monitored the expression of the gene represented by BI71N2, which we named *OsDR10* (*Oryza sativa* defense-responsive gene 10). *OsDR10* is an intronless gene and this gene and its homologs occur in the *Oryza* and *Leersia* genera of rice tribe, suggesting that it may have originated in the rice tribe. Suppressing *OsDR10* mediated a broad-spectrum resistance to *Xoo* accompanying the activation of the SA-dependent pathway and suppression of the jasmonic acid (JA)-dependent pathway, suggesting that OsDR10 is a negative regulator of rice disease resistance. These data provided the first opportunity to examine how one of the most important plant traits – disease resistance – evolved with the origination of a young new gene.

## Results

### Expression of *OsDR10* in response to pathogens and phytohormones

**Figure 1:**
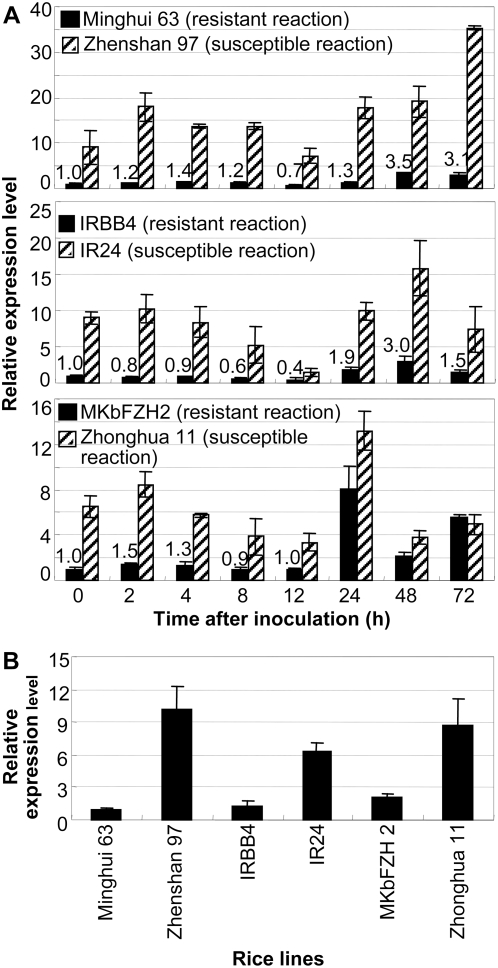
*OsDR10* expression in resistant and susceptible rice lines analyzed by qRT-PCR. Samples were collected before (0 h) and at 2, 4, 8, 12, 24, 48, and 72 h after pathogen (PXO61) inoculation. Each sample was from 3 to 5 plants. Bars represent mean (three technical replicates)±standard deviation. (A) Pathogen infection influenced *OsDR10* expression in both resistant and susceptible reactions in rice lines Minghui 63, Zhenshan 97, IRBB4, IR24, MkbFZH2, and Zhonghua 11. Figures indicate the expression level of *OsDR10* in resistant rice lines. (B) The relative expression levels of *OsDR10* in different rice lines.

Asian cultivated rice (*Oryza sativa* L.; AA genome) consists of two major groups, which are known by the subspecies names *indica* (*Oryza sativa* ssp. *indica*) and *japonica* (*O. sativa* ssp. *japonica*). To assess the effect of pathogens on *OsDR10* expression, quantitative reverse transcription (qRT)-PCR was applied to examine the transcript level of *OsDR10* in six different rice lines inoculated with *Xoo* strain PXO61. The *indica* cultivar Minghui 63, carrying two bacterial blight resistance genes, *Xa3/Xa26* and *Xa25(t)*, shows moderate resistance to PXO61 and *indica* cultivar Zhenshan 97, without any known *R* gene, is highly susceptible to PXO61 [19]. IRBB4, carrying bacterial blight resistance gene *Xa4*, is a near-isogenic line with the genetic background of the susceptible *indica* variety IR24 and is highly resistant to PXO61 [20]. MKbFZH2 is a transgenic line that overexpresses *Xa3/Xa26* in the genetic background of susceptible *japonica* cultivar Zhonghua 11 and is highly resistant to PXO61 [21]. The expression of *OsDR10* showed a similar pattern in both resistant and susceptible rice lines on pathogen infection (Figure 1A). *OsDR10* expression was first suppressed at 12 h in all rice lines except MKbFZH2 and then increased 1.8- to 8.1-fold at 24 to 72 h after PXO61 infection as compared to the corresponding uninfected plants. However, the expression level of *OsDR10* in susceptible rice lines was obviously higher than that in resistant rice lines, with or without pathogen infection. Comparative analysis of the expression levels of *OsDR10* in different rice lines showed that this gene had a similar expression level in the three resistant rice lines (Figure 1B), whereas the expression level in the three susceptible lines was approximately 3- to 10-fold higher than that in the resistant lines. These results suggest that a low expression level of *OsDR10* is associated with disease resistance.

In comparison with untreated plants, wounding significantly suppressed (*P*<0.05) *OsDR10* expression at 30 to 120 min after treatment (Figure S1). The phytohormones ethylene, JA, and SA, which are involved in pathogen-induced defense responses, also influenced the expression of *OsDR10*. Ethylene and SA first slightly induced and then suppressed *OsDR10* expression, and JA suppressed *OsDR10* expression as compared to the water (also as wounding) control. This result further suggests that *OsDR10* is involved in rice–pathogen interactions.

### Suppression of *OsDR10* enhances resistance to bacterial blight

Comparative analysis of the genomic and cDNA sequences of *OsDR10* (GenBank accession no. FJ194952) from *indica* cultivar Minghui 63 showed that *OsDR10* was an intronless gene consisting of 617 nucleotides and encoding an unknown protein of 100 amino acids (Figure S2A). Approximately half (49%) of the amino acid residues that compose the OsDR10 protein are charged (Figure S3). Structural analysis of OsDR10 provided no clue as to the mode of action of the protein.

**Figure 2:**
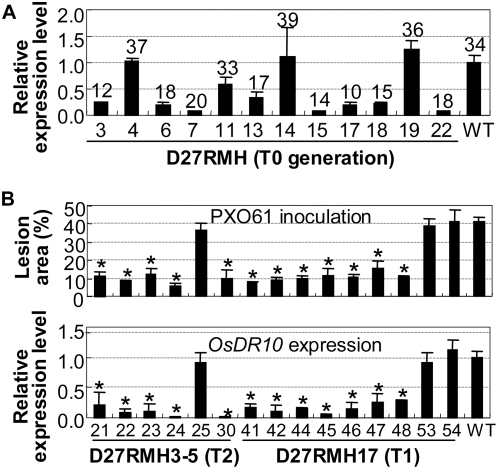
Enhanced resistance of *OsDR10*-suppressed plants to *Xoo* strain PXO61 was associated with suppressed *OsDR10* expression. Minghui 63 is wild type (WT). Bars represent mean (three technical replicates)±standard deviation. (A) T_0_ transgenic plants with significantly enhanced (*P*<0.01) bacterial resistance showed significantly reduced (*P*<0.01) accumulation of *OsDR10* transcripts. Figures indicate the lesion area caused by PXO61 infection. 0 d, samples were collected at 30 min after inoculation. (B) The enhanced resistance cosegregated with suppressed expression of *OsDR10* in T_1_ (D27RMH17) and T_2_ (D27RMH3-5) families. Asterisks indicate a significant difference in lesion area (*P*<0.01) or in *OsDR10* expression level (*P*<0.05) between transgenic and wild-type plants.

To determine whether *OsDR10* had a phenotypic impact on rice disease resistance, the RNA interference (RNAi) strategy was used to suppress the expression of *OsDR10* in Minghui 63, which is moderately resistant to *Xoo* strain PXO61 and susceptible to *Xoo* strain PXO99 [19]. Twenty-three independent transgenic plants were obtained. Each T_0_ transgenic plant was divided into two parts by separating the tillers at the tillering stage. The two parts were inoculated with PXO61 and PXO99, respectively, at the booting stage. Thirteen of the 23 plants showed significantly enhanced resistance to the two bacterial stains (*P*<0.01; Table S1). The lesion area of the 13 plants ranged from 10% to 24%, compared with 34% for the wild type after PXO61 infection, and from 16% to 45%, compared with 60% for the wild type after PXO99 infection (Table S1). The reduced lesion area of transgenic plants was highly correlated (*r* = 0.918, *α* = 0.01, *n* = 13 for PXO61 infection; *r* = 0.892, *α* = 0.01, *n* = 13 for PXO99 infection) with suppressed accumulation of *OsDR10* transcripts; transgenic plants showing enhanced resistance had 3.1- to 12.5-fold lower *OsDR10* transcripts than the wild-type plants (Figure 2A and Table S1).

To verify that the enhanced resistance of the transgenic plants was due to suppression of *OsDR10*, three T_1_ families generated from independent resistant T_0_ plants carrying a single copy of RNAi construct, D27RMH3, D27RMH15, and D27RMH17, were examined individually at the booting stage for resistance to PXO61 and the presence of the RNAi construct. All the T_1_ plants showing significantly enhanced resistance (*P*<0.05) to PXO61 carried the RNAi construct, whereas other plants showing no significant difference (*P*>0.05) from wild type in response to PXO61 infection were free of the RNAi construct (Table S2). A T_2_ family from a resistant T_1_ plant D27RMH3-5 and a T_1_ family from a resistant T_0_ plant D27RMH17 were further analyzed at the booting stage for resistance to PXO61 and expression of *OsDR10*. The enhanced resistance cosegregated with the reduced *OsDR10* transcripts in the T_1_ and T_2_ families (Figure 2B). The reduced lesion area of the transgenic plants was highly correlated with reduced *OsDR10* transcripts (*r* = 0.918, *α* = 0.01, *n* = 16). These results suggest that the enhanced bacterial resistance of the transgenic plants is due to suppressed expression of *OsDR10*.

**Figure 3:**
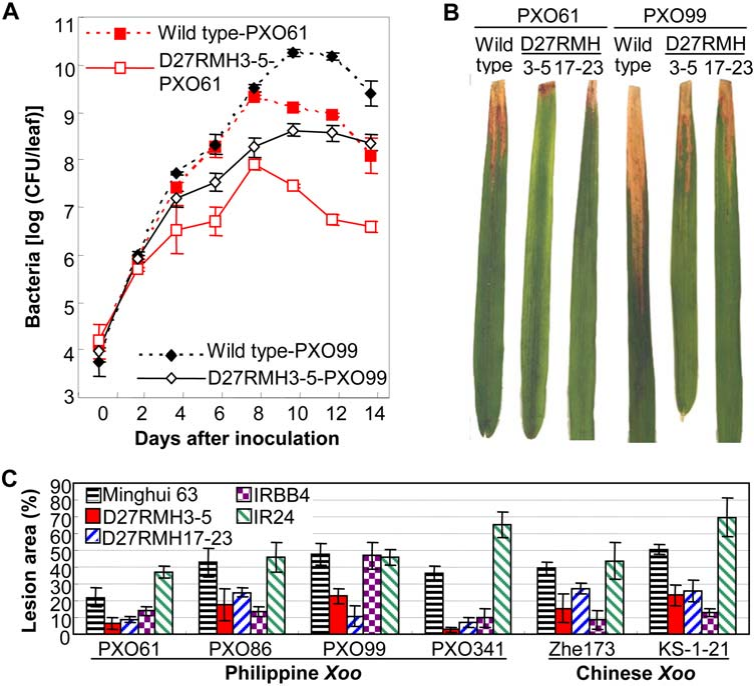
*OsDR10*-suppressed plants showed enhanced resistance to *Xoo* at both adult and seedling stages. PXO61, PXO86, PXO99, PXO341, Zhe173, and KS-1-21 were different *Xoo* strains that had different levels of virulence to the wild-type plant Minghui 63. D27RMH3-5 and D27RMH17-23 are transgenic lines of T_2_ generation. Each data point (A) or bar (C) represents mean (three technical replicates)±standard deviation. (A) Growth of *Xoo* strains PXO61 and PXO99 in leaves of *OsDR10*-suppressed and wild-type plants at booting stage. Bacterial populations were determined from three leaves at each time point by counting colony-forming units (CFUs). (B) *OsDR10*-suppressed plants showed enhanced bacterial resistance at the seedling stage. (C) *OsDR10*-suppressed plants showed broad-spectrum resistance to different *Xoo* strains at the booting stage. IRBB4, with bacterial blight resistance gene *Xa4*, is a near-isogenic line with the genetic background of the susceptible *indica* variety IR24.

The growth of *Xoo* strains PXO61 and PXO99 on resistant transgenic plants was 5- to 167-fold and 3- to 42-fold lower than that on corresponding wild-type control at 4 to 14 d after inoculation, respectively (Figure 3A). *OsDR10*-suppressed plants showed enhanced disease resistance not only at the adult stage but also at the seedling stage. Two T_2_ lines (D27RMH3-5 and D27RMH17-23) from two independent T_0_ plants were inoculated with PXO61 at the 5- or 6-leaf stage. They also showed enhanced resistance to *Xoo* as evaluated by lesion area (Figure 3B). The two transgenic lines were further examined for their resistant spectrum to different *Xoo* strains representing different races. Pathogen inoculation analysis demonstrated that these *OsDR10*-suppressed plants were also resistant to different *Xoo* strains (Figure 3C). Compared with the resistant rice line IRBB4, which carries the bacterial blight resistance gene *Xa4*, *OsDR10*-suppressed plants showed better resistance to *Xoo* strain PXO99 and similar resistance levels to strains PXO61, PXO86, and PXO341.

### Suppression of *OsDR10* affects transcript levels of a set of defense-responsive genes

**Figure 4:**
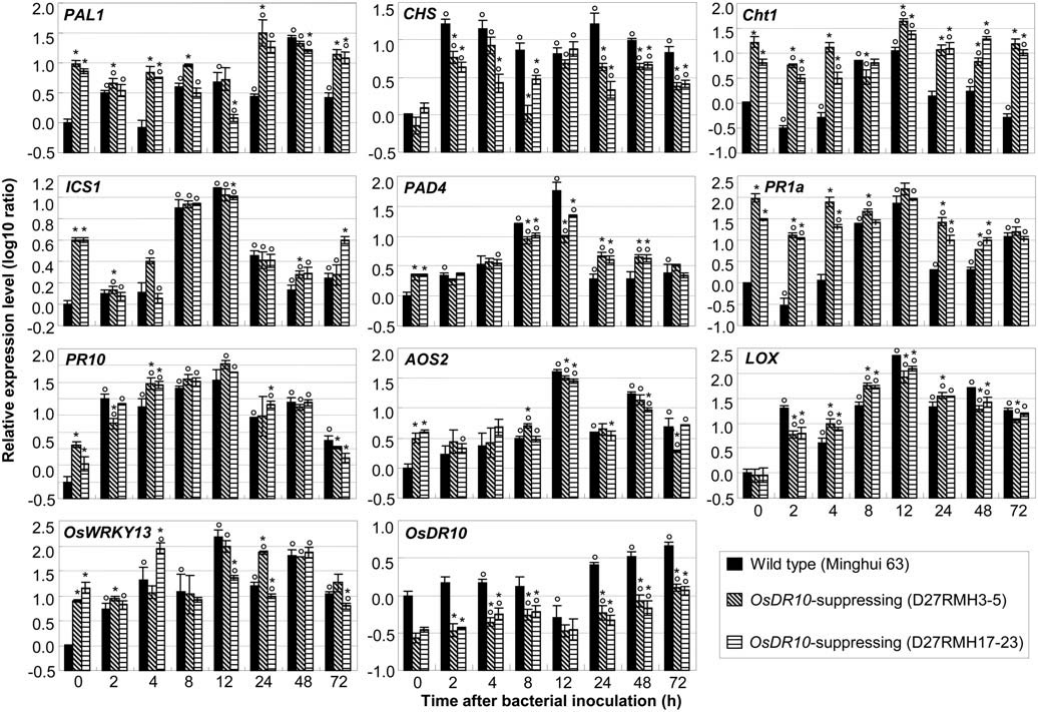
Expression analysis of defense-responsive genes in *OsDR10*-suppressed plants by qRT-PCR. Samples were collected before (0 h) and after inoculation of *Xoo* strain PXO61. Each sample was from 5 to 8 plants. Bars represent mean (three technical replicates)±standard deviation. Circles indicate a significant difference (*P*<0.05) between noninoculated and inoculated plants and asterisks indicate a significant difference (*P*<0.05) between the *OsDR10*-suppressed plants and corresponding wild-type plants within the same treatment.

The transcript levels of 12 defense-responsive genes, including those known to function in JA- and SA-dependent pathways, were examined in *OsDR10*-suppressed lines (D27RMH3-5 and D27RMH17-23) and wild-type Minghui 63 at the booting stage. Ten of the 12 genes showed differential expression either with or without pathogen infection (Figure 4). The 10 genes showed five types of expression patterns, considering the repetition of the two *OsDR10*-suppressed lines. *PAL1* (phenylalanine ammonia-lyase 1, GenBank accession no. X16099), *Cht1* (chitinase 1, D16221), and *PR1a* (acidic pathogenesis-related protein, AJ278436) showed 7- to 98-fold and 2- to 67-fold higher transcript levels in *OsDR10*-suppressed plants than in wild-type plants before and after bacterial infection, respectively. Compared to the wild-type plants, the transcript level of *PAD4* (phytoalexins deficient 4, CX118864) was 2-fold higher without pathogen infection and approximately 2-fold higher at some time points and 2- to 6-fold lower at other points after pathogen infection in *OsDR10*-suppressed plants. The expression of *ICS1* (isochorismate synthase 1, AK120689), *AOS2* (allene oxide synthase 2, AY062258), *PR10*, and *OsWRKY13* (EF143611) was approximately 2- to 15-fold higher in *OsDR10*-suppressed than in wild-type plants without bacterial infection, but there was a less than 2-fold difference in the two types of plants after bacterial infection. The transcript level of *CHS* (chalcone synthase, X89859) showed no significant difference (*P*>0.05) in *OsDR10*-suppressed and wild-type plants before bacterial infection, but it was 2- to 8-fold lower in *OsDR10*-suppressed plants than in wild-type plants after bacterial infection. The transcript level of *LOX* (lipoxygenase, D14000) was also not influenced (*P*>0.05) before bacterial infection, but was 2-fold higher at some time points and 2- to 3-fold lower at other points after pathogen infection in *OsDR10*-suppressed plants than in wild-type plants. The expression of *NH1* (*Arabidopsis* NPR1 homologue 1, AY923983) and *OsMPK6* (EF174189) showed no marked difference in *OsDR10*-suppressed and wild-type plants.

### Suppression of *OsDR10* enhances the SA level and suppresses JA level

**Figure 5:**
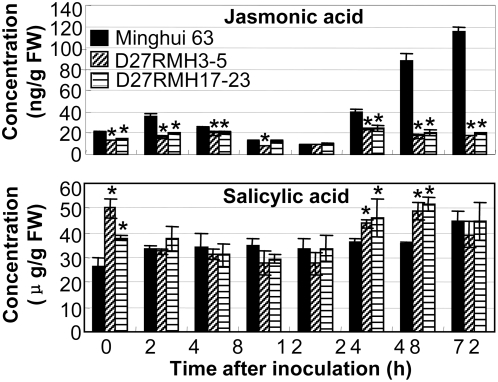
Concentration of jasmonic acid and salicylic acid. Jasmonic acid and salicylic acid levels in the leaves of *OsDR10*-suppressed lines (D27RMH3-5 and D27RMH17-23) and wild-type plants (Minghui 63) were measured before (0 h) and after inoculation of *Xoo* strain PXO61. FW, fresh weight. Each sample was from 5 to 8 plants. Each bar represents mean (three technical replicates)±standard deviation.

To examine whether the modified expression of defense-responsive genes caused by *OsDR10* influences the endogenous levels of JA and SA, we quantified the concentrations of the two signal molecules in the leaves of *OsDR10*-suppressed T_2_ lines. Pathogen infection significantly induced (*P*<0.05) JA accumulation in both *OsDR10*-suppressed plants (maximum 1.8-fold) and wild-type plants (maximum 5.7-fold; Figure 5). However, JA concentration was significantly lower (*P*<0.05) in *OsDR10*-suppressed plants both before (approximately 1.5-fold) and at 72 h (5.8- to 6.5-fold) after bacterial infection than that in wild-type plants. Pathogen infection only slightly influenced SA accumulation in *OsDR10*-suppressed and wild-type plants (Figure 5), but SA concentration was significantly higher (*P*<0.05) in *OsDR10*-suppressed plants both before (1.4- to 1.9-fold) and at 24 to 48 h (1.2- to 1.5-fold) after bacterial infection than that in wild-type plants. The concentration of conjugated SA, SA β-glucoside, was not influenced by the suppressed expression of *OsDR10* (data not shown).

### *OsDR10* and its homologs are only detected in the species of rice tribe

There is one homolog (named *OsDR10-NipponbareA* and *OsDR10-9311A* in this paper) of *OsDR10* in the genomes of *japonica* rice cultivar Nipponbare and *indica* 93-11 based on BLAST analysis against the whole genome sequences of the two cultivars [22], suggesting that *OsDR10* is member of a small gene family in *O. sativa* (Table 1). A cDNA harboring the complete coding region of *OsDR10-NipponbareA* was identified to support its expression in Nipponbare (Table 1). In addition, another *OsDR10*-homologous cDNA (*OsDR10-NipponbareB*) from Nipponbare and another *OsDR10*-homologous genomic sequence (*OsDR10-9311B*) from 93-11, which putatively encode a same protein of 105 amino acids, were identified from GenBank (Table 1 and Figure S3). Several *OsDR10* homologous cDNA or genomic sequences were also identified from *japonica* rice cultivar Nackdong, different accessions of common wild rice *O. rufipogon* (AA genome), and wild rice *O. australiensis* (EE genome) by BLAST analysis (Table 1). These *OsDR10* homologous genes putatively encode proteins consisting of 99 to 106 amino acids (Figure S3).

**Table 1:** *OsDR10* gene and its homologs in different plant species.

Gene name	Source material	Sequence type	GenBank accession no.	Sequence identity (%)^a^	Length of homolog sequence (nt)^b^	Protein size (aa)
*OsDR10*	Minghui 63 (*O. sativa* ssp. *indica*)	genomic	FJ194952			100
*OsDR10-9311A*	93-11 (*O. sativa* ssp. *indica*)	genomic	AAAA02023082	100/100	617	100
*OsDR10-NipponbareA*	Nipponbare (*O. sativa* ssp. *japonica*)	cDNA	CI254543	100/100	609	100
*OsDR10-O.rufipogonA*	*O. rufipogon*	BAC end	CL821758	97/98	606	99
*OsDR10-O.rufipogonB*	*O. rufipogon*	cDNA	CU861690	98/98	597	99
*OsDR10-9311B*	93-11	genomic	AAAA02023080	92/93	630	105
*OsDR10-NipponbareB*	Nipponbare	cDNA	CI242307	91/93	609	105
*OsDR10-Nackdong*	Nackdong (*O. sativa* ssp. *japonica*)	cDNA	CF325892	92/93	528	105
*OsDR10-O.punctata*	*O. punctata*	genomic	FJ528577	69/69	285	95
*OsDR10-O.latifolia*	*O. latifolia*	genomic	FJ528578	69/69	294	98
*OsDR10-O.australiensis*	*O. australiensis*	BAC end	CL931253	77/76	510	106
*OsDR10-L.tisserantii*	*Leersia tisserantii*	genomic	FJ528579	69/69	315	105
*OsDR10-L.JX*	*Leersia tisserantii* (Jiangxi)	genomic	FJ528580	73/73	303	101

a Identity to *OsDR10* gene. The two numbers represent the identity of whole homologous region/identity to the coding region of *OsDR10*.

b Length of sequence homologous to *OsDR10* gene.

*OsDR10* and its product have no discernible sequence similarity to the genes and proteins from organisms other than rice, according to BLAST analysis of the nucleotide database GenBank and the protein database of the National Center for Biotechnology Information. However, BLAST analyses showed that sequences flanking *OsDR10* were homologous to the sequences of other species. A 3490-nt sequence, located upstream of *OsDR10* and 9912 nt away from *OsDR10* and a 620-nt gene (*Os08g05970*), located immediately downstream of *OsDR10* and 998 nt away from *OsDR10* based on TIGR annotation, were homologous to the sequences of monocots maize and dicots *Medicago truncatula* (barrel medic) (Table 2). These results suggest that *OsDR10* may be a rice tribe-specific gene.

**Table 2:** Sequences homologous to *OsDR10* flanking sequences.

Sequence source	GenBank accession no.	Length of homologous *OsDR10* flanking sequence	Identity (%)	E value
Maize clone CH201-134E23	AC187899	80% of 3490-nt^a^	82 to 86	0 to 7e-33
Barrel medic clone mth2-47e6	CR932965	58% of 3490-nt^a^	73 to 82	5e-149 to 2e-28
Maize clone CH201-82D4	AC214434	145 nt of 620-nt^b^	87	2e-49

a Sequence located upstream of *OsDR10*.

b Sequence located downstream of *OsDR10*.

**Figure 6:**
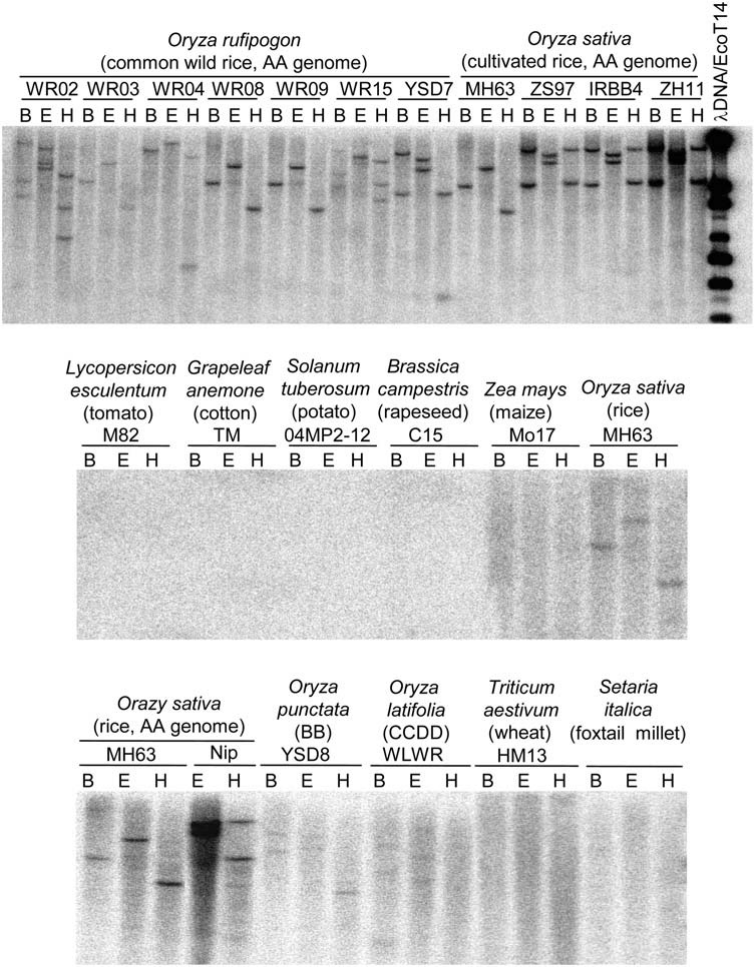
DNA gel blot analysis of *OsDR10*-homologous genes in different plant species. WR02, WR03, WR04, WR08, WR09, WR15, and YSD7 are different common wild rice accessions. MH63 (Minghui 63), ZS97 (Zhenshan 97), and IRBB4 are *indica* rice lines, and ZH11 (Zhonghua 11) and NIP (Nipponbare) are *japonica* rice lines. B, *Bam*HI; E, *Eco*RI; H, *Hin*dIII.

To test this hypothesis, DNA gel blot analysis was performed to examine homologous sequences of *OsDR10* in different plant species. The analysis showed that *OsDR10* occurred in cultivated rice, common wild rice (*O. rufipogon*), and other wild rice, including *O. punctata* (BB genome), and *O. latifolia* (CCDD genome; Figure 6 and Figure S4). One to three hybridization bands were detected for the DNA from various rice accessions digested with different restriction enzymes under high-stringency hybridization. No homologous sequence was detected in other cereal species (maize, wheat, and foxtail millet) or dicot crops (cotton, potato, tomato, and rapeseed) under low-stringency DNA hybridization (Figure 6 and Figure S4). These results suggest that *OsDR10* does not occur in the species outside of rice tribe Oryzeae and this gene has sequence diversity or a copy number difference in various rice species.

We also used PCR sequencing to detect *OsDR10* homologs from the species that were tested by DNA gel blot analyses and their closer relatives. The beginning and end sequences of the coding regions of the sequences listed above are conserved (Figure S5). The conserved regions were used to design PCR primers to amplify *OsDR10* homologs from different species of Poaceae (grass) family. Clear PCR product bands were detected after electrophoresis in other two wild rice species, *O. punctate* and *O. latifolia*, of *Oryza* genus, two *Leersia* species, *L. tisserantii* and *L.* JX , of *Leersia* genus, and the *Bambusoideae* species, bamboo, but not in wheat, barley, foxtail millet, maize, and sorghum. The *Oryza* and *Leersia* genera belonging to rice tribe Oryzeae and *Leersia* is the closest relatives of *Oryza* genus [23]. Sequencing analysis of these PCR products showed that *O. punctuate*, *O. latifolia*, *L. tisseranti*, and *L.* JX but not bamboo contained *OsDR10* homologs (Table 1 and Figure S5). The sequences from bamboo matched with only the primer sequences but not with *OsDR10* (data not shown). These analyses further support the conclusion from the DNA gel blot and BLAST analyses: *OsDR10* is a rice tribe-specific gene that may be generated by *de novo* evolution.

### The evolution of *OsDR10* was conservative

**Figure 7:**
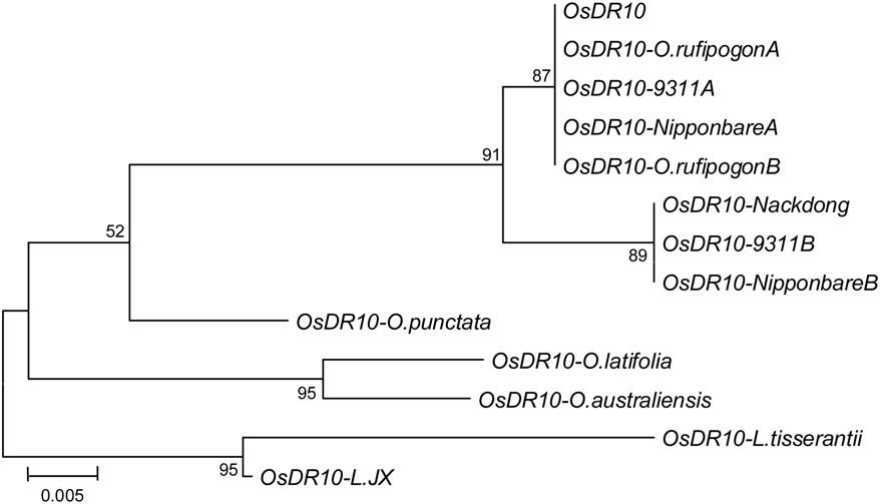
Phylogenetic analysis of the coding regions of *OsDR10* and its homologs from different species. The tree was constructed by the neighbor-joining method. The numbers for interior branches indicate the bootstrap values (%) for 1000 replications. The scale at the bottom is in units of number of nucleotide substitutions per site.

The *OsDR10* gene and its homologs in different species of *Oryza* and *Leersia* genera share 67% to 100% sequence identity each other in the coding region (Table S3). These genes putatively encode nine types of proteins (Figure S3) that share 55% to 99% sequence identity and 57% to 99% sequence similarity with each other (Table S4). Phylogenetic analysis of the coding regions of these genes showed the similar evolution relationship as reported previously, in which the *Leersia* genus is more closely related to *O. australiensis* than to other species of the *Oryza* genus examined (Figure 7) [23]. Thus *OsDR10* from rice line Minghui 63 has the most distant phylogenetic relationship with *Leersia* in the coding region. The ratio of nonsynonymous (Ka) to synonymous (Ks) substitution is a measure of natural selection acting on the protein. If there is no selective pressure (neutral evolution) on a gene, the ratio will be approximately 1. A Ka/Ks ratio <1 indicates functional constraint with purifying selection on the gene, and a Ka/Ks ratio >1 indicates accelerated evolution with positive selection. The Ka and Ks of the coding sequences of *OsDR10* and *OsDR10-L.JX* are 0.1331 and 0.3361, respectively, for a ratio of 0.3960 (*P* = 0.0126<0.05). Within the *Oryza* genus, *OsDR10* has the highest sequence diversity from *OsDR10-O .punctata*. The Ka and Ks of the coding sequences of *OsDR10* and *OsDR10-O .punctata* are 0.1072 and 0.4199, respectively, for a ratio of 0.2553 (*P* = 0.0031<0.01). These results suggest that the evolution of *OsDR10* was conservative and *OsDR10* may implement important functions in rice.

## Discussion

### *OsDR10* is a rice tribe-specific gene

*OsDR10* is a new gene and may have originated *de novo*. This hypothesis is supported by the following evidence. First, *OsDR10* or its homologs were detected only in *Oryza* species and *Leersia* species, the closest relative of *Oryza* genus, based on sequencing, BLAST and DNA gel blot analyses, whereas the sequences homologous to the flanking sequences of *OsDR10* were identified in other plant species. Furthermore, the close relative of rice tribe, the Bambusoideae subfamily [24], did not carry *OsDR10* homologs. These results suggest that *OsDR10* may originate in the rice tribe and is a new rice-specific gene. Second, the predicted OsDR10 protein and its homologs do not have sequence homology with any known, expressed, or hypothetical proteins. Third, *OsDR10* is a single-exon gene, and it has been reported that the coding region of recently evolved *de novo* genes in *D. melanogaster* are not interrupted by intron [8].

An alternative interpretation with the *de novo* origination would be that the rapid sequence evolution of this gene could have lost identity with the homologous genes, if they exist, in non-*Oryza* species. We tested this hypothesis by considering the age of *Oryza* and the age of maize. The age of the rice tribe is around ∼14 million years [25], [26]. Given the average substitution rate for grasses (5.9×10^−9^ substitutions per synonymous site per year) [26]–[28] under the assumption of the neutral constant substitution process, the divergence at synonymous sites in the evolutionary time of 2×14 million years would be 16.5% between the rice genes and the hypothetical homologs in the grass branches from the most recent ancestors of the rice genus. This study showed that the replacement site evolved 2.5 to 3.9 times (3.2 of the median) lower than synonymous sites; thus, the divergence at the replacement would be 16.5/3.2 = 5.2%. The divergence of the entire coding region would be expected to be 8% (the weighted average of the divergence at the two types of sites). Very conservatively, we can take maize as the most recent outgroup that does not have any homologs of *OsDR10*, which diverged from rice 50 million years ago [29]. A similar estimation led to an expected divergence of 28% between maize and rice in the entire gene region. Even using the fastest known rate of substitution for grasses (7.0×10^−9^ substitutions per synonymous site per year) [28] to test this hypothesis, the expected divergence between maize and rice in the entire gene region is 67%. Thus, we should have been able to detect the homologous sequences in maize with this level of similarity. But we did not observe this homologous sequence using the all three methods: the genomic Southern analysis, the BLAST analysis of the sequenced genomes of maize, and the PCR amplification, in contrast to an expectation that can be made given the highly constraint detected from this gene. Therefore, we rejected this alternative hypothesis of identity loss in evolution, in strong support of the conclusion of the *de novo* origination.

Our Southern DNA gel blot analysis suggests that there are one to three *OsDR10* homologous genes in different rice varieties and wild rice accessions. Rice variety Minghui 63 and some common wild rice accessions contain only one copy of *OsDR10*, whereas other rice varieties, including Nipponbare, and wild rice accessions may contain two or three copies of *OsDR10* homologs. This suggestion can be supported by DNA sequence analysis. The homologous rice genes of *OsDR10* have been identified based on BLAST analysis. The sequences of *OsDR10* and its homologous genes in different rice accessions do not contain the digestion sites of restriction enzymes used in our DNA gel blot analysis.

### OsDR10 is a suppressor of the NH1-uninvolved SA-dependent pathway and an activator of the JA-dependent pathway

Although several whole genes of *de novo* origination have been identified outside plants, the roles of these genes in molecular regulation are largely unknown. It has been reported that spermatogenesis appears to be the functional target for newly evolved genes in the lineages of *Drosophila*. Several *de novo* genes have been reported to show male-specific expression in these species [8]–[11]. The *de novo* gene *BSC4* may be involved in the DNA repair pathway during the stationary phase of *S. cerevisiae* [12].

Our results suggest that OsDR10 is a negative regulator of rice defense responses against bacterial infection. Low expression level of *OsDR10* is associated with bacterial resistance. Both *R* genes and *Xoo* influence *OsDR10* expression. In the same genetic background, rice lines carrying an *R* gene showed a significantly lower level of *OsDR10* transcripts than those without an *R* gene, suggesting that OsDR10 may be negatively involved in some *R* gene-mediated defense signaling transduction. In contrast, *OsDR10* expression was induced by *Xoo*, indicating that OsDR10 is a potential target of pathogen infection.

JA and SA are important signaling molecules in induced disease resistance of rice [30], [31]. Our results showed that the enhanced disease resistance of *OsDR10*-suppressed plants was accompanied by increased accumulation of SA and suppressed accumulation of JA, suggesting that OsDR10 is a negative regulator of SA synthesis and a positive regulator of JA synthesis. SA can be synthesized via both the isochorismate pathway and phenylpropanoid pathway in *Arabidopsis* and putatively in rice as well [31]–[33]. ICS1 and PAD4 are involved in SA synthesis in the isochorismate pathway [34]. PALs belong to a large family and are key enzymes for SA synthesis in the phenylpropanoid pathway. OsWRKY13 is an activator of ICS1 and PAD4 in rice disease resistance [31]. OsDR10 appears to inhibit the expression of *OsWRKY13*, *ICS1*, *PAD4*, and *PAL1* when free of pathogen infection and only inhibits the expression of *PAL1* after pathogen infection. However, OsDR10 is an activator of CHS, which functions on a branch of the phenylpropanoid pathway and catalyzes synthesis of defensive secondary metabolites using the intermediate of SA synthesis as a matrix [35]. Our results suggest that OsDR10 inhibits SA synthesis, mainly via the phenylpropanoid pathway during defense responses. *PR1a* functions in an SA-dependent pathway in rice bacterial resistance [31]. The increased *Cht1* expression in *OsDR10*-suppressed plants was associated with accumulation of SA, suggesting that it may also function in an SA-dependent pathway. *PR1a* and *Cht1* showed a similar expression pattern in both *OsDR10*-suppressed and wild-type plants, suggesting that the two genes function in the same SA-dependent pathway, which is suppressed by OsDR10. NH1 is both the sequence and functional ortholog of *Arabidopsis* NPR1, which is a key regulator of SA-dependent systemic acquired resistance [36] and functions downstream of OsMPK6 in bacterial blight resistance [37]. The expression of *NH1* and *OsMPK6* was not influenced by suppressed expression of *OsDR10*. Thus, the SA-dependent pathway suppressed by OsDR10 is an NH1-independent pathway. LOX and AOS are two important enzymes in the biosynthesis of JA [30]. The reduced accumulation of JA in *OsDR10*-suppressed plants was associated with suppressed expression of LOX and AOS2 after bacterial infection, suggesting that OsDR10 is an activator of the JA-dependent pathway and it functions upstream of JA.

### *OsDR10* has been functionally constrained during evolution

During the evolution of a genome, those genes required for or that facilitate the survival of a species are created and maintained, whereas unwanted genes are discarded. As a negative regulator of rice disease resistance, *OsDR10* appears to be important for rice. This hypothesis is supported by the following evidence. First, *OsDR10* originated after cereal crops diverged and the evolution of *OsDR10* was conservative. Plant disease resistance signaling pathways consist of both active and negative factors. Elimination of the functions of negative regulators of disease resistance frequently results in abnormal plants [37]–[39], which suggests that functional balance of the activities of the two types of factors in disease resistance is essential for the normal activity of plants. OsDR10 contributes to this balance. Second, the open reading frame of OsDR10 has been maintained during evolution. At least six different sizes (95, 99, 100, 101, 105, and 106 amino acid residues) of OsDR10 protein and its homologs were identified. Comparative alignment of the coding regions of the *OsDR10* gene and its homologs showed that all the deletions and insertions of the genes during evolution occurred in triplet or multiple triplets in one site, which did not shift the original reading frames (Figure S5). For example, 75 deletions/insertions in 12 sites were observed in the coding region of *OsDR10-O. australiensis* compared to that of *OsDR10*, but all the deletions/insertions showed reading frame maintenance. In comparison, 17 deletion/insertion sites were detected in the 3′- and 5′-untranslated regions of *OsDR10* homologs compared to those of *OsDR10*, but 15 of the 17 sites showed deletion/insertion with one, two, four, five, seven, or eight nucleotides (Figure S6).

In conclusion, our study identified a novel gene of *de novo* origination in rice tribe. The encoding product of this gene functions as a negative regulator in the SA-dependent pathway to balance rice defense response induced by pathogen infection. These results provide fresh insights into the new biologic and evolutionary processes of a *de novo* gene recruited rapidly in plant. The present data also put *OsDR10* as a tentative “orphan” gene for it is unknown how this gene appeared in the rice tribe. Further studies on the identification of low homologous sequences of *OsDR10* from the other species of rice tribe may help us to disclose how this gene was generated, when the genomic sequence of all the species in rice tribe are available. Further studies of tissue-specific expression pattern of *OsDR10* in the entire life cycle of rice will help us target the putative function of this gene in other physiologic or developmental processes in addition to disease resistance.

## Materials and Methods

### Gene isolation and structural analysis

BI71N2 is the cDNA fragment of *OsDR10* from the cultivated rice Minghui 63 (*O. sativa* ssp. *indica*) normalized cDNA library [17]. The sequence of BI71N2 was used to screen the GenBank nucleotide sequence database (http://www.ncbi.nlm.nih.gov) and Rice EST (expression sequence tag) DataBase (REDB, http://redb.ncpgr.cn) for homologous genomic and cDNA sequences using the BLAST program [22]. The analysis indicated that homologous cDNA sequence EI87G08 from Minghui 63 is the full-length cDNA of *OsDR10*. To predict the structure of *OsDR10*, the homologous genomic sequence of EI87G08 was analyzed using the GenScan program (http://genes.mit.edu/GENSCAN.html). *OsDR10* was isolated from Minghui 63 by PCR amplification using primers 87G8-F2 (5′-CAGAATTCAACTTTTATCACACGTTTAACG-3′) and 87G8-R3 (5′-ACGGATCCTTCATCATCGTCATCCTC-3′) designed based on the sequences flanking *OsDR10*. The Minghui 63 *OsDR10* sequence was confirmed by sequencing analysis using primers 87G8-F2, 87G8-R3, and 87G8-R1 (5′-ACGGATCCTTCATCATCGTCATCCTC-3′'; underlined, *Bam*HI digestion site).

PCR amplification of *OsDR10* homologous from different grass species was performed using PCR primers 87G8-R1 and 87G8-R2 (5′-AAGGTACCATGGCGTTCTACAAGTACGG-3′; underlined, *Kpn*I digestion site) with different annealing temperatures to obtain PCR products. The PCR products were excided from agarose gel after electrophoresis and purified for sequencing. Each PCR product was sequenced at least three times. The consensus sequences were used for further analysis.

### Pathogen inoculation

To examine the resistance of plants to bacterial blight disease, plants were inoculated with Philippine *Xoo* strain PXO61 (race 1) at the seedling stage and four Philippine *Xoo* strains, PXO61, PXO86 (race 2), PXO99 (race 6), and PXO341 (race 10), and two Chinese *Xoo* strains, Z173 and K-S-21, at the booting stage by the leaf clipping method [40]. Disease was scored (3–5 leaves for each plant) as the percent lesion area (lesion length/leaf length) at 2 to 3 weeks after inoculation. The bacterial population in rice leaves was determined by counting colony-forming units [19].

### Hormone treatments

Phytohormone treatments were applied according to the procedures described previously [31]. Leaf segments approximately 2-cm long were cut from the fully expanded leaves of 21-d Minghui 63 and floated on 30 mL of solution containing 100 µM of SA, JA, or ethephon (an ethylene generator) in covered sterile Petri dishes. Leaf segment wounding by cutting and floating on deionized water served as a control.

### RNA expression analyses

Aliquots (15 µg) of total RNA were used for RNA gel blot analysis [31]. A 617-bp probe of *OsDR10* digested from cDNA clone EI87G08 with restriction enzymes *Kpn*I and *Bam*HI was used for the hybridization. qRT-PCR was conducted using gene-specific primers (Table S5) as described previously [31]. The assays were repeated at least twice, with each repetition having three replicates; similar results were obtained in repeated experiments. Standard deviation was calculated for technical replicates.

### Rice transformation

The RNAi vector of *OsDR10* was constructed by inserting a fragment of cDNA digested from cDNA clone BI71N2 using restriction enzymes *Kpn*I and *Bam*HI into the pDS1301 vector (Figure S2B) [37]. The recombinant plasmid was introduced into *Agrobacterium tumefaciens* strain EHA105 by electroporation. *Agrobacterium*-mediated transformation was performed using calli derived from mature embryos of Minghui 63 according to a published protocol [19].

### Quantification of SA and JA

The leaves of rice plants at the booting stage were used to quantify endogenous SA and JA. The SA and conjugated SA samples were prepared and quantified using high-performance liquid chromatography as described previously [31]. The JA samples were prepared as described previously [6], with some modifications. The leaf samples were purified and diluted with 300 µL methanol using the method described by Ding et al [6]. Derivatization of JA using ethereal diazomethane was accessional. Ethereal diazomethane was synthesized from N-nitrosomethyl urea following the method of Müller et al [41]. Ethereal diazomethane (150 µL) was added to the methanol redissolved samples for derivatization at room temperature for 15 min, followed by the addition of 40 µL 0.2 M acetic acid/methanol to counteract the excessive diazomethane. The samples were dried by evaporation with nitrogen gas at 35°C. Samples were dissolved in 30 µL hexane and a 1-µL aliquot of the sample was injected into the gas chromatograph/mass spectrometer system (GCMS-QP2010S, Shimadzu, Japan) under the conditions of 77°C for 1.2 min, 10°C/min to 180°C, and 20°C/min to 250°C. The quantitative data of JA and the internal standard dihydro-JA were obtained using the peak of the product ion 151 with retention times 12.613 min and 12.680 min, respectively.

### DNA gel blot analysis

Rice total DNA was digested with *Bam*HI, *Eco*RI, and *Hin*dIII, separated by electrophoresis on 0.8% agarose gels, and blotted onto nylon membranes. The cDNA of *OsDR10* was used as probe for hybridization. DNA filters were hybridized at 55°C overnight and subjected to two low-stringency washes (10 min at room temperature and 5 min at 55°C) with a solution composed of 2×SSC and 0.1% SDS. The same filters were also hybridized at 65°C overnight and subjected to two high-stringency washes (10 min at room temperature and 5 min at 65°C) with a solution composed of 2×SSC and 0.1% SDS. The hybridization signals were detected using a fluorescent image analyzing system.

### Analysis of gene evolution

Multiple-sequence alignment of the coding sequences of *OsDR10* and its homologs was performed using the ClustalX program [42]. The Ka and Ks were determined from the alignments with a modified Nei-Gojobori model in MEGA4 and the difference between Ka and Ks was tested using the Z-test embedded in MEGA4 [43].

## Supporting Information

Figure S1 The effects of defense signal molecules on the expression of OsDR10. OsDR10 expression was analyzed by qRT-PCR in the leaves of rice cultivar Minghui 63. Each sample was from 10 to 20 plants. Bars represent mean (three technical replicates)±standard deviation. Asterisks indicate a significant difference (P<0.05) between the hormone treatment and the water (also as wounding) control, which also served as wounding treatment for the leaves were cut from plants and treated in vitro, at the same time point.
(1.96 MB TIF)

Figure S2 Schematic diagrams of the OsDR10 gene and its transformation construct. (A) OsDR10 gene. The coding region (black box), 5′- and 3′-untranslated regions (hatched boxes), translation start codon (ATG), and translation stop codon (TAG) are also indicated. The numbers indicate the base pairs of each substructure. (B) RNAi construct of OsDR10. RB and LB, right and left T-DNA border; GUS, β-glucuronidase gene; Hpt, hygromycin phosphotransferase gene; 35S, cauliflower mosaic virus 35S promoter; OCS, octopine synthase polyadenylation signal.
(1.88 MB TIF)

Figure S3 Alignment of OsDR10 protein sequence with its homologs. Dash indicates a gap.
(2.03 MB TIF)

Figure S4 DNA gel blot analysis of OsDR10-homologous gene in different plant species. MH63 (Minghui 63) is indica rice line and NIP (Nipponbare) is japonica rice line. B, BamHI; E, EcoRI; H, HindIII. (A) and (B) No OsDR10 homologous sequence was detected in plants other than rice. Rice 5s rDNA probe amplified using primers 5srDNAF (5′-GGATGC GATCATACCAGCAC-3′) and 5srDNAR (5′- GGGATGCAACACAAGGACTTC-3′) was used to examine the quality of DNA for DNA gel blot analysis.
(1.76 MB PDF)

Figure S5 Alignment of OsDR10 coding sequence with its homologs. The sequences of OsDR10-O.punctat, OsDR10-O. lafifolia, OsDR10-L. tisserantii, and OsDR10-L. JX were obtained by PCR amplification followed by sequencing the PCR products. The locations of PCR primers in the coding region are underlined. Dash indicates a gap.
(0.12 MB PDF)

Figure S6 Aignment of the 5′- and 3′-untranslated regions of OsDR10 gene and its homologs. Dash indicates a gap.
(3.17 MB TIF)

Table S1 Performance of T0 OsDR10-suppressed plants (D27RMH) to Xoo strains PXO61 and PXO99
(0.08 MB PDF)

Table S2 Cosegregation analysis of enhanced resistance to Xoo strain PXO61 and existence of the RNAi construct in OsDR10-suppressed T1 families
(0.13 MB PDF)

Table S3 Sequence identity (%) among OsDR10 gene and its homologs from different species
(0.09 MB PDF)

Table S4 Sequence identity/similarity (%) among OsDR10 protein and its homologs in different species
(0.08 MB PDF)

Table S5 Gene-specific primers for qRT-PCR
(0.09 MB PDF)
