# Supplementary figures and images for: A Rice Gene of De Novo Origin Negatively Regulates Pathogen-Induced Defense Response
Source: PLoS One. 2009 Feb 25;4(2):e4603. doi: 10.1371/journal.pone.0004603 (PMC2643483; doi:10.1371/journal.pone.0004603)

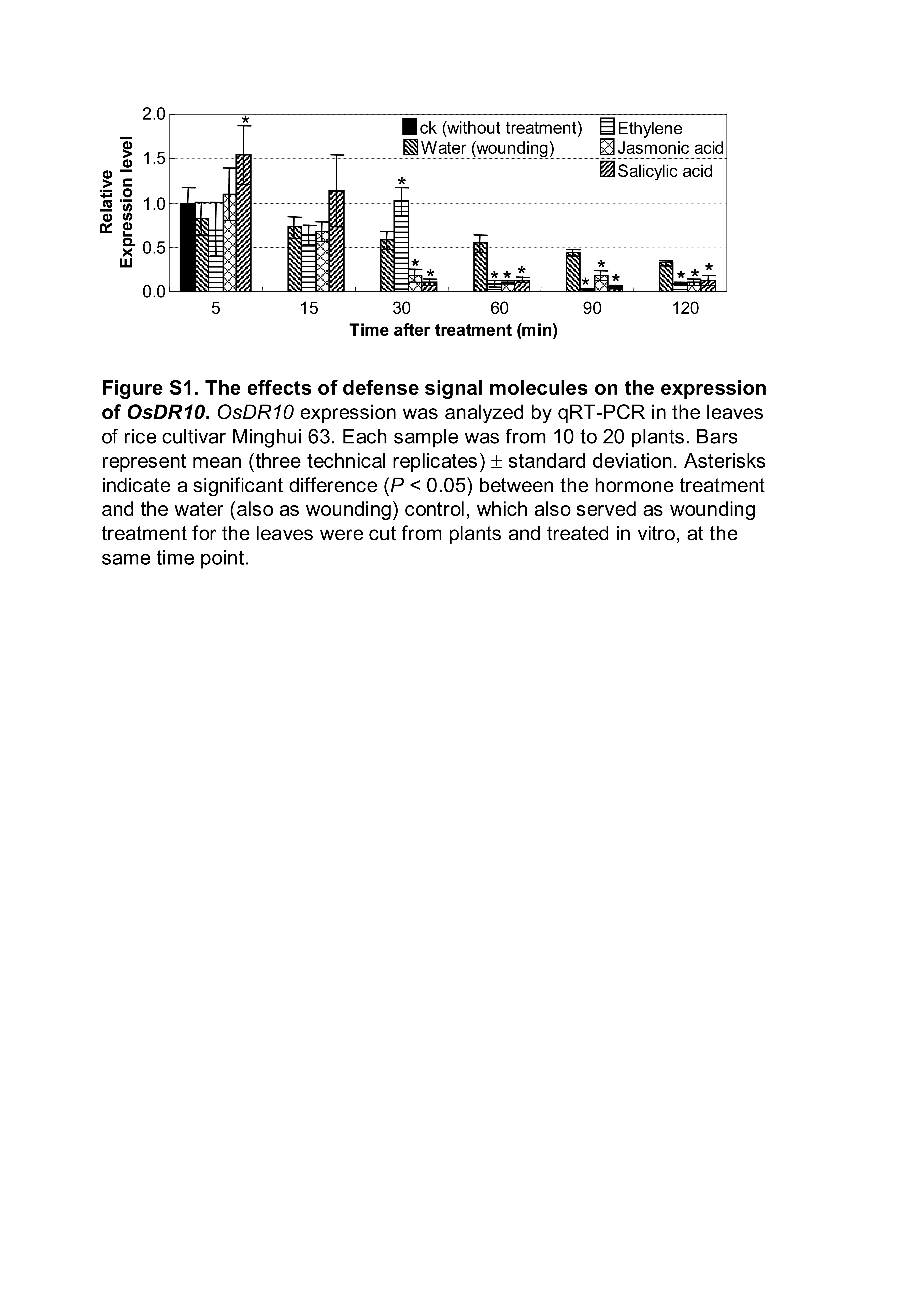

Supplement: Figure S1 — The effects of defense signal molecules on the expression of OsDR10. OsDR10 expression was analyzed by qRT-PCR in the leaves of rice cultivar Minghui 63. Each sample was from 10 to 20 plants. Bars represent mean (three technical replicates)±standard deviation. Asterisks indicate a significant difference (P<0.05) between the hormone treatment and the water (also as wounding) control, which also served as wounding treatment for the leaves were cut from plants and treated in vitro, at the same time point. (1.96 MB TIF) [file pone.0004603.s001.tif]

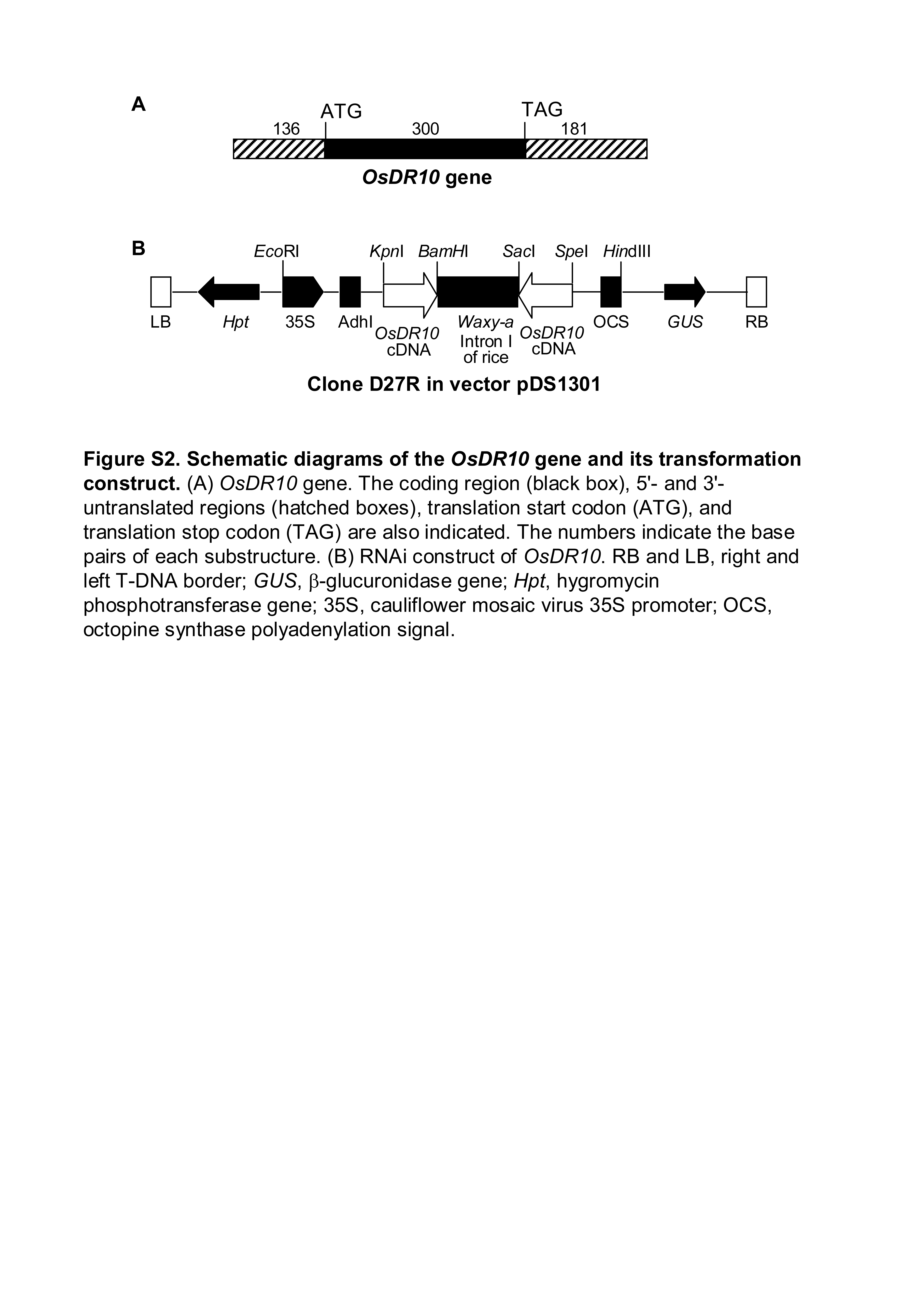

Supplement: Figure S2 — Schematic diagrams of the OsDR10 gene and its transformation construct. (A) OsDR10 gene. The coding region (black box), 5′- and 3′-untranslated regions (hatched boxes), translation start codon (ATG), and translation stop codon (TAG) are also indicated. The numbers indicate the base pairs of each substructure. (B) RNAi construct of OsDR10. RB and LB, right and left T-DNA border; GUS, β-glucuronidase gene; Hpt, hygromycin phosphotransferase gene; 35S, cauliflower mosaic virus 35S promoter; OCS, octopine synthase polyadenylation signal. (1.88 MB TIF) [file pone.0004603.s002.tif]

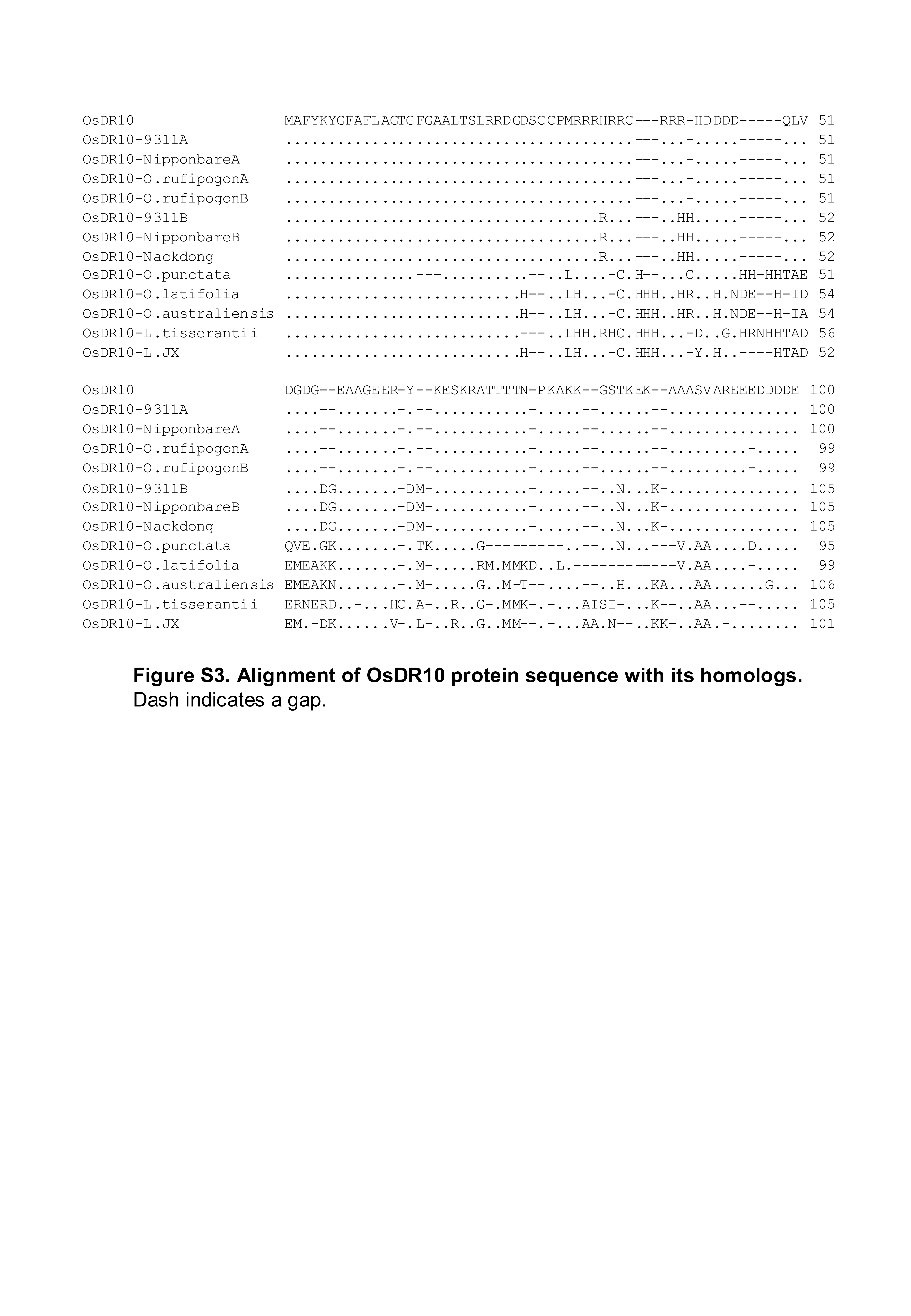

Supplement: Figure S3 — Alignment of OsDR10 protein sequence with its homologs. Dash indicates a gap. (2.03 MB TIF) [file pone.0004603.s003.tif]

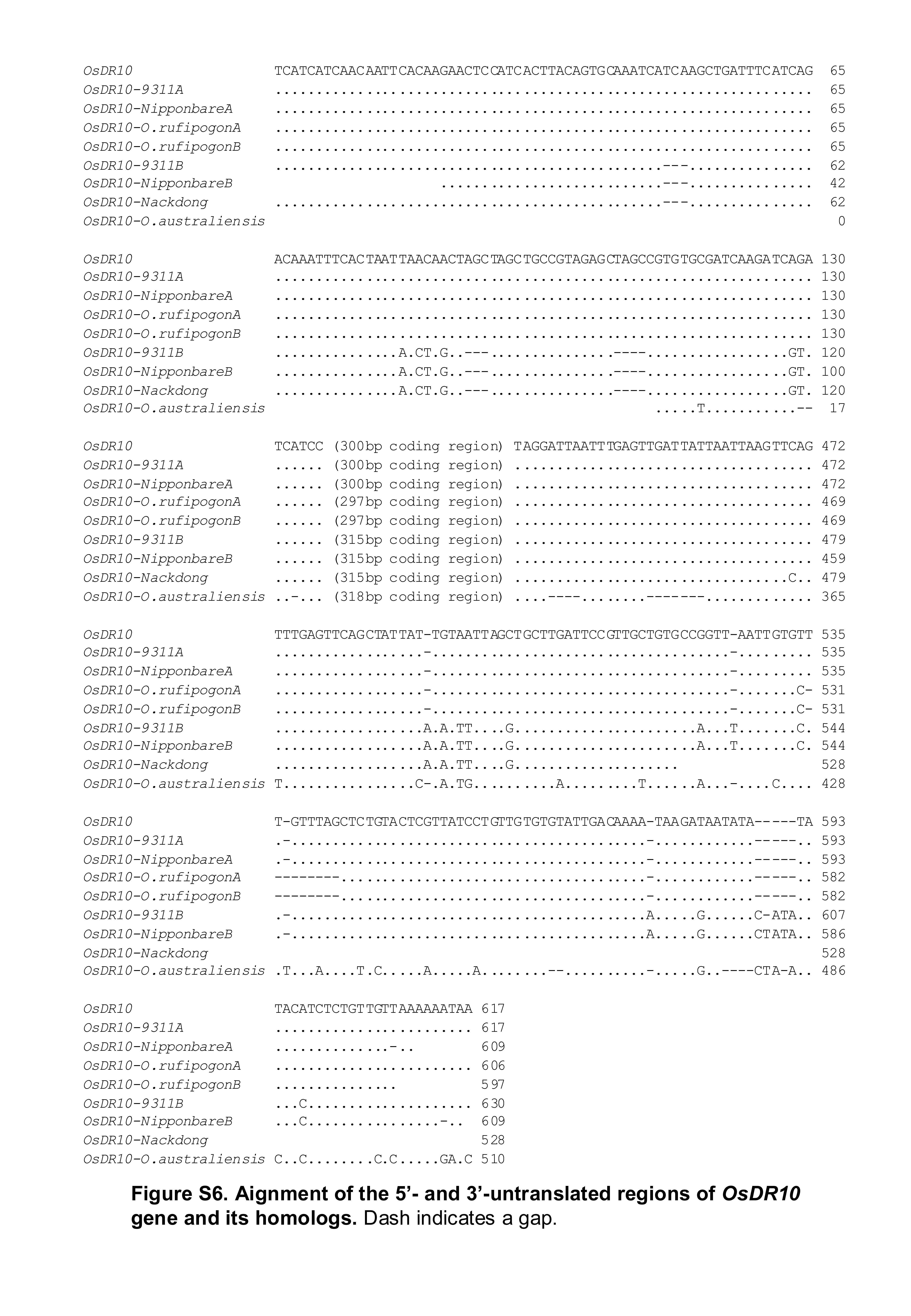

Supplement: Figure S6 — Aignment of the 5′- and 3′-untranslated regions of OsDR10 gene and its homologs. Dash indicates a gap. (3.17 MB TIF) [file pone.0004603.s006.tif]
