# Supplementary material for: A Rice Gene of De Novo Origin Negatively Regulates Pathogen-Induced Defense Response
Source: PLoS One. 2009 Feb 25;4(2):e4603. doi: 10.1371/journal.pone.0004603 (PMC2643483; doi:10.1371/journal.pone.0004603)
Supplement: Figure S4 — DNA gel blot analysis of OsDR10-homologous gene in different plant species. MH63 (Minghui 63) is indica rice line and NIP (Nipponbare) is japonica rice line. B, BamHI; E, EcoRI; H, HindIII. (A) and (B) No OsDR10 homologous sequence was detected in plants other than rice. Rice 5s rDNA probe amplified using primers 5srDNAF (5′-GGATGC GATCATACCAGCAC-3′) and 5srDNAR (5′- GGGATGCAACACAAGGACTTC-3′) was used to examine the quality of DNA for DNA gel blot analysis. (1.76 MB PDF) [file pone.0004603.s004.pdf]

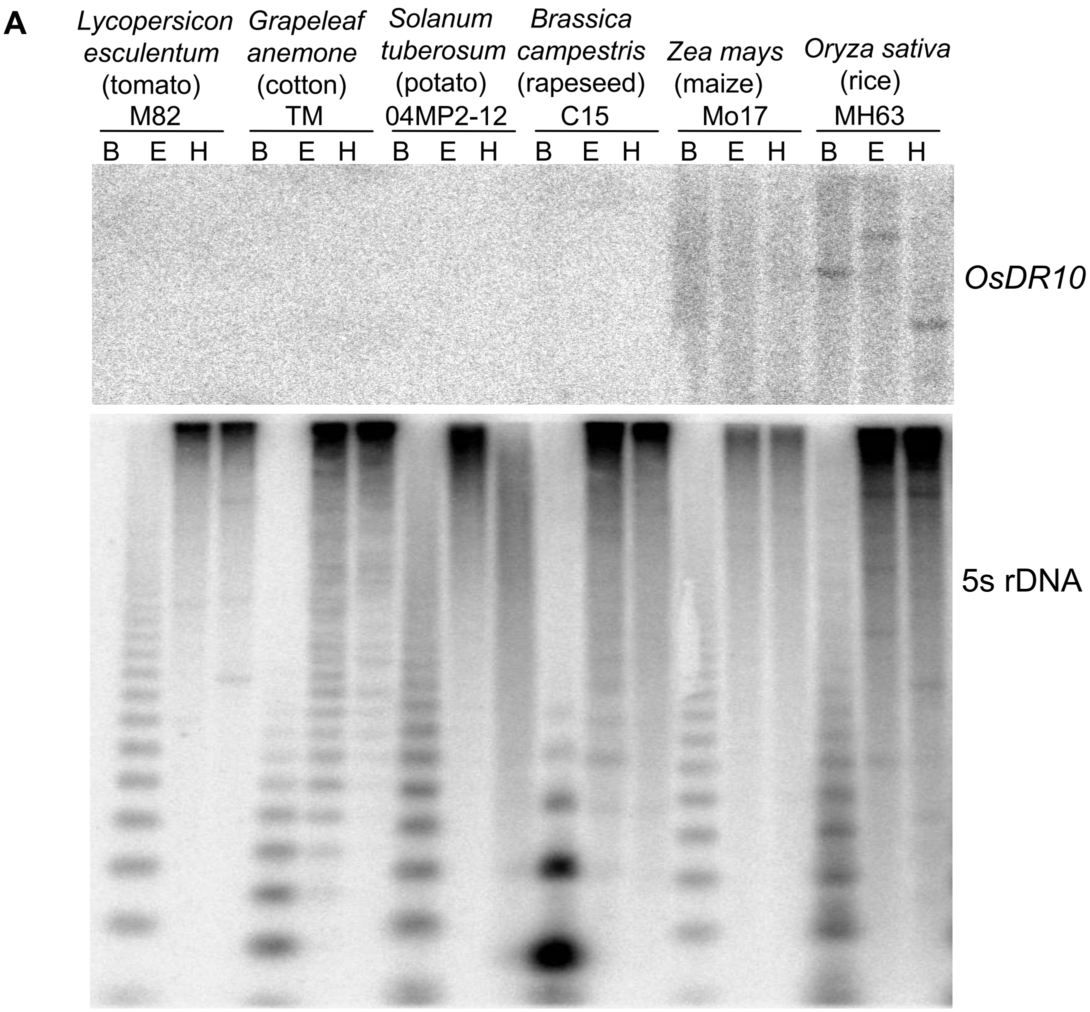
A
Lycopersicon esculentum
(tomato)
M82
Grapeleaf anemone
(cotton)
TM
Solanum tuberosum
(potato)
04MP2-12
Brassica campestris
(rapeseed)
C15
Zea mays
(maize)
Mo17
Oryza sativa
(rice)
MH63
B E H B E H B E H B E H B E H B E H
OsDR10
5s rDNA

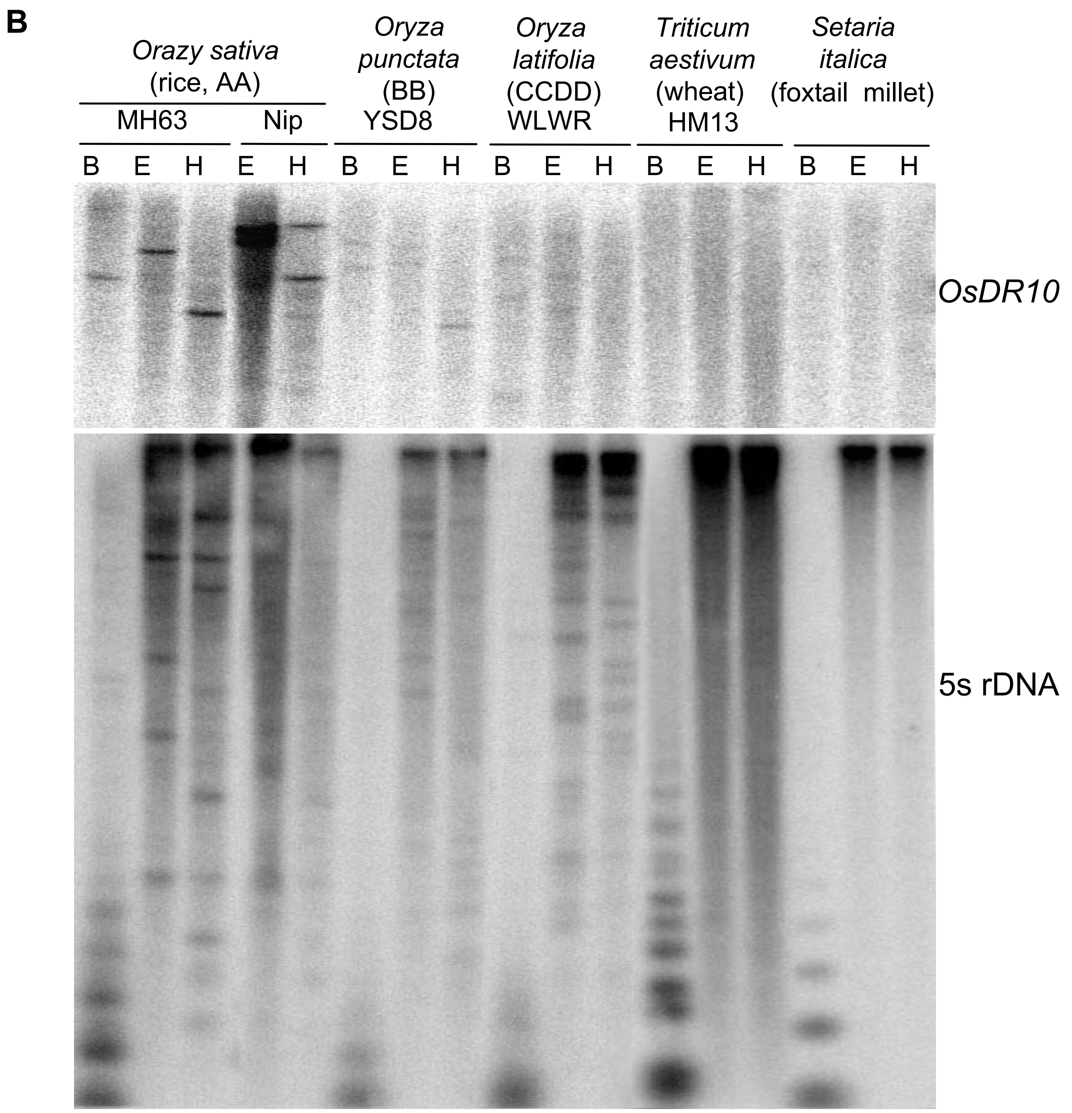

Figure S4. DNA gel blot analysis of *OsDR10*-homologous gene in different plant species. MH63 (Minghui 63) is *indica* rice line and NIP (Nipponbare) is *japonica* rice line. B, *Bam*HI; E, *Eco*RI; H, *Hin*dIII. (A) and **(**B) No *OsDR10* homologous sequence was detected in plants other than rice. Rice 5s rDNA probe amplified using primers 5srDNAF (5'-GGATGC GATCATACCAGCAC-3') and 5srDNAR (5'-GGGATGCAACACAAGGACTTC-3') was used to examine the quality of DNA for DNA gel blot analysis.
