# Supplementary material for: A Rice Gene of De Novo Origin Negatively Regulates Pathogen-Induced Defense Response
Source: PLoS One. 2009 Feb 25;4(2):e4603. doi: 10.1371/journal.pone.0004603 (PMC2643483; doi:10.1371/journal.pone.0004603)
Supplement: Figure S5 — Alignment of OsDR10 coding sequence with its homologs. The sequences of OsDR10-O.punctat, OsDR10-O. lafifolia, OsDR10-L. tisserantii, and OsDR10-L. JX were obtained by PCR amplification followed by sequencing the PCR products. The locations of PCR primers in the coding region are underlined. Dash indicates a gap. (0.12 MB PDF) [file pone.0004603.s005.pdf]

```
OsDR10                   ATG GCG TTC TAC AAG TAC GGC TTC GCC TTC TTG GCC GGC ACC GGC TTC GGC  51
OsDR10-9311A             ... ... ... ... ... ... ... ... ... ... ... ... ... ... ... ... ...  51
OsDR10-NipponbareA       ... ... ... ... ... ... ... ... ... ... ... ... ... ... ... ... ...  51
OsDR10-O.rufipogonA      ... ... ... ... ... ... ... ... ... ... ... ... ... ... ... ... ...  51
OsDR10-O.rufipogonB      ... ... ... ... ... ... ... ... ... ... ... ... ... ... ... ... ...  51
OsDR10-9311B             ... ... ... ... ... ... ... ... ... ... ... ... ... ... ... ... ...  51
OsDR10-NipponbareB       ... ... ... ... ... ... ... ... ... ... ... ... ... ... ... ... ...  51
OsDR10-Nackdong          ... ... ... ... ... ... ... ... ... ... ... ... ... ... ... ... ...  51
OsDR10-O.punctata        ... ... ... ... ... ... ..G ... ... ... ... ... ... ... ... --- ---  45
OsDR10-O.latifolia       ... ... ... ... ... ... ..G ... ... ... ... ... ... ... ... ... ...  51
OsDR10-O.australiensis   ... ... ... ... ... ... ..G ... ..T ... ... ... ... ... ... ... ...  51
OsDR10-L.tisserantii     ... ... ... ... ... ... ... ... ... ... ... ... ... ... ... ... ...  51
OsDR10-L.JX              ... ... ... ... ... ... ..G ... ... ... ... ... ... ... ... ... ...  51

OsDR10                   GCC GCG CTC ACC AGC CTC CGC CGC GAC GGC GAC AGC TGC TGC CCC ATG CGC 102
OsDR10-9311A             ... ... ... ... ... ... ... ... ... ... ... ... ... ... ... ... ... 102
OsDR10-NipponbareA       ... ... ... ... ... ... ... ... ... ... ... ... ... ... ... ... ... 102
OsDR10-O.rufipogonA      ... ... ... ... ... ... ... ... ... ... ... ... ... ... ... ... ... 102
OsDR10-O.rufipogonB      ... ... ... ... ... ... ... ... ... ... ... ... ... ... ... ... ... 102
OsDR10-9311B             ... ... ... ... ... ... ... ... ... ... ... ... ... ... ... ... ... 102
OsDR10-NipponbareB       ... ... ... ... ... ... ... ... ... ... ... ... ... ... ... ... ... 102
OsDR10-Nackdong          ... ... ... ... ... ... ... ... ... ... ... ... ... ... ... ... ... 102
OsDR10-O.punctata        --- ... ..G ... ... ... ... ... ... ... ... --- --- ... ... C.C ...  87
OsDR10-O.latifolia       ... ... ..G ..G ... ... ... ... ... ... C.. --- --- ... ... C.C .AT  96
OsDR10-O.australiensis   ... ... ..G ..G ... ... ... ... ... ... C.. --- --- ... ... C.C .AT  96
OsDR10-L.tisserantii     ... ... ..G ... ... ... ... ... ... ... --- --- --- ... ... C.C .A.  93
OsDR10-L.JX              ... ... ..G ..G ... ... ... ... ... ... C.. --- --- ... ... C.C .AT  96

OsDR10                   CGC CGC CAC CGC CGC TGT --- --- --- CGC CGC CGC --- CAC GAC GAC GAC 141
OsDR10-9311A             ... ... ... ... ... ... --- --- --- ... ... ... --- ... ... ... ... 141
OsDR10-NipponbareA       ... ... ... ... ... ... --- --- --- ... ... ... --- ... ... ... ... 141
OsDR10-O.rufipogonA      ... ... ... ... ... ... --- --- --- ... ... ... --- ... ... ... ... 141
OsDR10-O.rufipogonB      ... ... ... ... ... ... --- --- --- ... ... ... --- ... ... ... ... 141
OsDR10-9311B             ... ... .G. ... ... ... --- --- --- ... ... .A. CAC ... ... ... ... 144
OsDR10-NipponbareB       ... ... .G. ... ... ... --- --- --- ... ... .A. CAC ... ... ... ... 144
OsDR10-Nackdong          ... ... .G. ... ... ... --- --- --- ... ... .A. CAC ... ... ... ... 144
OsDR10-O.punctata        ... ... ... --- T.. ..C CAC --- --- ... ... ..T TGT ... ... ... ... 129
OsDR10-O.latifolia       ... ... ... --- T.. ..C CAC CAC CAT ... ... .AT CGC ..T ... C.. ... 144
OsDR10-O.australiensis   ... ... ... --- T.. ..C CAC CAC CAT ... ... .AT CGC ..T ... C.. ... 144
OsDR10-L.tisserantii     .A. ..G .G. .AT T.. ..C CAC CAT CAC ... ... ... --- G.. ... ... .G. 141
OsDR10-L.JX              ... ... ... --- T.. ..C CAC CAC CAC ... ..T ... --- T.. ... C.. ... 141

OsDR10                   GAC --- --- --- --- --- CAG CTG GTC GAC GGC GAC GGC --- --- GAG GCT 171
OsDR10-9311A             ... --- --- --- --- --- ... ... ... ... ... ... ... --- --- ... ... 171
OsDR10-NipponbareA       ... --- --- --- --- --- ... ... ... ... ... ... ... --- --- ... ... 171
OsDR10-O.rufipogonA      ... --- --- --- --- --- ... ... ... ... ... ... ... --- --- ... ... 171
OsDR10-O.rufipogonB      ... --- --- --- --- --- ... ... ... ... ... ... ... --- --- ... ... 171
OsDR10-9311B             ... --- --- --- --- --- ... ... ... ... ... ... ... GAC GGC ... ... 180
OsDR10-NipponbareB       ... --- --- --- --- --- ... ... ... ... ... ... ... GAC GGC ... ... 180
OsDR10-Nackdong          ... --- --- --- --- --- ... ... ... ... ... ... ... GAC GGC ... ... 180
OsDR10-O.punctata        ... CAC CAC --- CAC CAC ACC GCC .AG C.G .TG ..G ..G GGC AAG ..A ... 177
OsDR10-O.latifolia       A.. GAC GAG --- --- CAT --- A.C .A. ..G ATG ..G .CG AAA AAA ... ... 186
OsDR10-O.australiensis   A.. GAC GAG --- --- CAC --- A.C .C. ..G ATG ..G .CG AAG AAC ... ... 186
OsDR10-L.tisserantii     ... CAC CGG AAT CAC CAC ACC GCC .A. ..G A.G A.T .AA AGG GAT ..A ... 192
OsDR10-L.JX              ... --- --- --- --- CAC ACC GCC .A. ..G ATG ..T --- GAC AAG ... ..A 177
```

```
OsDR10                  GCA GGG GAA GAG CGA --- TAC --- --- AAG GAG AGC AAG AGG GCG ACG ACG 213
OsDR10-9311A            ... ... ... ... ... --- ... --- --- ... ... ... ... ... ... ... ... 213
OsDR10-NipponbareA      ... ... ... ... ... --- ... --- --- ... ... ... ... ... ... ... ... 213
OsDR10-O.rufipogonA     ... ... ... ... ... --- ... --- --- ... ... ... ... ... ... ... ... 213
OsDR10-O.rufipogonB     ... ... ... ... ... --- ... --- --- ... ... ... ... ... ... ... ... 213
OsDR10-9311B            ... ... ... ... ... --- G.. ATG --- ... ... ... ... ... ... ... ... 225
OsDR10-NipponbareB      ... ... ... ... ... --- G.. ATG --- ... ... ... ... ... ... ... ... 225
OsDR10-Nackdong         ... ... ... ... ... --- G.. ATG --- ... ... ... ... ... ... ... ... 225
OsDR10-O.punctata       ... ... ..G ... ..C --- ... ACG AAG ... ... ... ... ... .GC --- --- 219
OsDR10-O.latifolia      ..G ... ..G ... ..C --- ... ATG --- ... ... ... ... ... AG. .T. ... 231
OsDR10-O.australiensis  ... ... ..G ... ..G --- ... ATG --- ... ... ... ... ..A .G. ... ... 231
OsDR10-L.tisserantii    --- ... ..G ... .AT TGT ... GCG --- ... ..A ..A ... ... .GA --- ... 234
OsDR10-L.JX             ..T ... ..G ... GTC --- ... TTG --- ... ... ..A ... ... .GA ... ... 222

OsDR10                  ACG ACG AAT --- CCC AAG GCC AAG AAG --- --- GGC AGC ACC AAG GAG AAG 255
OsDR10-9311A            ... ... ... --- ... ... ... ... ... --- --- ... ... ... ... ... ... 255
OsDR10-NipponbareA      ... ... ... --- ... ... ... ... ... --- --- ... ... ... ... ... ... 255
OsDR10-O.rufipogonA     ... ... ... --- ... ... ... ... ..A --- --- ... ... ... ... ... ... 255
OsDR10-O.rufipogonB     ... ... ... --- ... ... ... ... ... --- --- ... ... ... ... ... ... 255
OsDR10-9311B            ... ... ... --- ..G ... ... ... ..A --- --- ... ... .A. ... ... ... 267
OsDR10-NipponbareB      ... ... ... --- ..G ... ... ... ..A --- --- ... ... .A. ... ... ... 267
OsDR10-Nackdong         ... ... ... --- ..G ... ... ... ..A --- --- ... ... .A. ... ... ... 267
OsDR10-O.punctata       --- --- --- --- --- --- --- ... ..A --- --- ... ... .A. ... ... ... 243
OsDR10-O.latifolia      .T. .T. ..G GAT ..G ..A TTG ... --- --- --- --- --- --- --- --- --- 255
OsDR10-O.australiensis  .T. --- .CG --- --- ... ... ... ... --- --- ... ... CA. ... ... ... 267
OsDR10-L.tisserantii    .T. .T. ..G --- ..G --- ... ... ... GCA ATT A.. .T. --- ... ..A ... 276
OsDR10-L.JX             .T. .T. --- --- ..G --- ... ... ... GCT GCC ... .A. --- --- ... ... 258

OsDR10                  --- --- GCA GCT GCT AGT GTT GCT AGG GAG GAG GAG GAT GAC GAT GAT GAA 300
OsDR10-9311A            --- --- ... ... ... ... ... ... ... ... ... ... ... ... ... ... ... 300
OsDR10-NipponbareA      --- --- ... ... ... ... ... ... ... ... ... ... ... ... ... ... ... 300
OsDR10-O.rufipogonA     --- --- ... ... ... ... ... ... ... ... ... --- ... ... ... ... ... 297
OsDR10-O.rufipogonB     --- --- ... ... ... ... ... ... ... ... ... --- ... ... ... ... ... 297
OsDR10-9311B            AAG --- ... ... ... ... ... ... ... ... ... ... ... ... ... ... ... 315
OsDR10-NipponbareB      AAG --- ... ... ... ... ... ... ... ... ... ... ... ... ... ... ... 315
OsDR10-Nackdong         AAG --- ... ... ... ... ... ... ... ... ... ... ... ... ... ... ... 315
OsDR10-O.punctata       --- --- --- .TA ... GCA .C. ..C ... ... ... ..T ..C ..T ... ... ... 285
OsDR10-O.latifolia      --- --- --- .TC ... GCG .C. ... ..A ... ... --- ... ... ... ... ... 297
OsDR10-O.australiensis  AAG GCA ..T ..A ... GCA .C. ... ... ... ... ... ... .G. ... ... ... 318
OsDR10-L.tisserantii    AAG --- --- ..A ... GCA .C. ..C ... ... --- --- ... ... ... ... ... 315
OsDR10-L.JX             AAG AAG --- ... ... GC. .C. ..G --- ... ... ... ... ... ... ... ... 303
```

Figure S5. Alignment of *OsDR10* coding sequence with its homologs. The sequences of *OsDR10-O.punctat*, *OsDR10-O. lafifolia*, *OsDR10-L. tisserantii*, and *OsDR10-L*. JX were obtained by PCR amplification followed by sequencing the PCR products. The locations of PCR primers in the coding region are underlined. Dash indicates a gap.
