# Supplementary material for: A Rice Gene of De Novo Origin Negatively Regulates Pathogen-Induced Defense Response
Source: PLoS One. 2009 Feb 25;4(2):e4603. doi: 10.1371/journal.pone.0004603 (PMC2643483; doi:10.1371/journal.pone.0004603)
Supplement: Table S1 — Performance of T0 OsDR10-suppressed plants (D27RMH) to Xoo strains PXO61 and PXO99 (0.08 MB PDF) [file pone.0004603.s007.pdf]

Table S1. Performance of T_0 *OsDR10*-suppressed plants (D27RMH) to *Xoo* strains PXO61 and PXO99

Rice material	PXO61		PXO99	
	Lesion area (%)[a]	*P*[c]	Lesion area (%)[a]	*P*[c]
D27RMH2	32.0 ± 4.9	0.5313	56.3 ± 6.6	0.3593
D27RMH3	12.3 ± 5.7	0.0000	22.7 ± 3.5	0.0000
D27RMH4	36.6 ± 5.9	0.3287	60.1 ± 6.2	0.9382
D27RMH6	18.3 ± 7.4	0.0012	33.4 ± 8.5	0.0000
D27RMH7	20.1 ± 5.9	0.0017	31.3 ± 6.0	0.0000
D27RMH8	23.6 ± 3.2	0.0069	42.7 ± 3.8	0.0016
D27RMH9	22.6 ± 3.2	0.0030	29.7 ± 9.9	0.0000
D27RMH10	13.6 ± 3.8	0.0000	29.5 ± 8.2	0.0000
D27RMH11	32.5 ± 3.6	0.5817	55.0 ± 8.9	0.2501
D27RMH12	23.2 ± 7.2	0.0089	45.0 ± 4.9	0.0078
D27RMH13	17.4 ± 3.2	0.0000	21.5 ± 8.9	0.0000
D27RMH14[b]	39.4 ± 5.9	0.0649	55.4 ± 7.2	0.2592
D27RMH15	14.4 ± 5.2	0.0000	19.7 ± 6.2	0.0000
D27RMH17	9.8 ± 4.6	0.0000	16.3 ± 3.4	0.0000
D27RMH18	14.7 ± 3.5	0.0000	30.7 ± 10.0	0.0000
D27RMH19[b]	36.3 ± 2.5	0.3155	54.9 ± 5.7	0.1978
D27RMH20	37.8 ± 4.9	0.1546	60.4 ± 5.7	0.8762
D27RMH21	36.3 ± 4.8	0.3613	59.5 ± 10.5	0.9405
D27RMH22	17.6 ± 4.1	0.0000	22.1 ± 5.6	0.0000
D27RMH23	34.4 ± 4.8	0.8436	60.1 ± 7.9	0.9505
D27RMH24	32.0 ± 5.4	0.4948	53.2 ± 10.7	0.1530
D27RMH25	14.5 ± 7.8	0.0000	22.8 ± 6.2	0.0000
D27RMH26[b]	35.1 ± 2.6	0.6277	60.9 ± 7.2	0.7733
Minghui63 (wild type)	33.8 ± 4.9		59.8 ± 7.2	

[a]For most of the plants, four to five uppermost fully expanded leaves of each plant were inoculated. The lesion area was recoded at 21 d after bacterial inoculation.

[b]The negative transgenic plants detected by PCR analysis.

[c]Each *P* value was calculated in comparison with wild type.
