# Supplementary material for: A Rice Gene of De Novo Origin Negatively Regulates Pathogen-Induced Defense Response
Source: PLoS One. 2009 Feb 25;4(2):e4603. doi: 10.1371/journal.pone.0004603 (PMC2643483; doi:10.1371/journal.pone.0004603)
Supplement: Table S2 — Cosegregation analysis of enhanced resistance to Xoo strain PXO61 and existence of the RNAi construct in OsDR10-suppressed T1 families (0.13 MB PDF) [file pone.0004603.s008.pdf]

Table S2. Cosegregation analysis of enhanced resistance to *Xoo* strain PXO61 and existence of the RNAi construct in *OsDR10*-suppressed T_1 families

Rice material	Lesion area (%)[a]	P^b	RNAi construct[c]
D27RMH3 T_1 family			
D27RMH3-1	27.7 ± 2.6	0.1650	−
D27RMH3-2	21.1 ± 5.8	0.0033	+
D27RMH3-3	14.7 ± 4.2	0.0000	+
D27RMH3-4	19.2 ± 3.3	0.0000	+
D27RMH3-5	7.7 ± 1.3	0.0000	+
D27RMH3-6	20.6 ± 4.2	0.0004	+
D27RMH3-7	20.3 ± 3.1	0.0003	+
D27RMH3-8	20.8 ± 6.2	0.0031	+
D27RMH3-9	27.3 ± 3.2	0.1295	−
D27RMH3-10	19.6 ± 3.7	0.0004	+
D27RMH3-11	15.3 ± 3.1	0.0000	+
D27RMH3-12	23.7 ± 7.5	0.0924	−
D27RMH3-14	14.1 ± 4.4	0.0352	+
D27RMH3-15	17.9 ± 2.2	0.0001	+
D27RMH3-17	14.1 ± 4.9	0.0000	+
D27RMH3-21	27.2 ± 4.6	0.4051	−
D27RMH3-22	8.2 ± 2.2	0.0000	+
D27RMH3-23	27.5 ± 5.8	0.4476	−
D27RMH3-24	9.2 ± 2.8	0.0000	+
D27RMH3-25	21.2 ± 2.2	0.0253	+
D27RMH15 T_1 family			
D27RMH15-21	24.7 ± 4.8	0.1557	−
D27RMH15-22	10.4 ± 6.1	0.0001	+
D27RMH15-23	17.2 ± 4.0	0.0033	+
D27RMH15-24	6.9 ± 5.2	0.0003	+
D27RMH15-25	19.7 ± 5.6	0.0148	+
D27RMH15-26	23.1 ± 1.4	0.0670	−

D27RMH15-27	7.4 ± 5.0	0.0000	+
D27RMH15-28	17.3 ± 2.8	0.0032	+
D27RMH17 T_1 family			
D27RMH17-21	26.1 ± 6.0	0.2799	–
D27RMH17-22	7.9 ± 3.6	0.0000	+
D27RMH17-23	3.7 ± 2.1	0.0001	+
D27RMH17-24	4.2 ± 2.0	0.0001	+
D27RMH17-25	7.1 ± 7.4	0.0000	+
D27RMH17-26	3.0 ± 1.5	0.0000	+
D27RMH17-27	5.8 ± 3.8	0.0000	+
D27RMH17-28	7.3 ± 1.8	0.0000	+
D27RMH17-29	2.5 ± 0.9	0.0000	+
D27RMH17-30	4.3 ± 1.5	0.0000	+
D27RMH17-31	24.5 ± 1.5	0.1282	–
D27RMH17-32	9.0 ± 4.6	0.0002	+
D27RMH17-33	17.2 ± 5.2	0.0038	+
D27RMH17-34	17.1 ± 7.4	0.0048	+
D27RMH17-35	1.9 ± 0.3	0.0000	+
D27RMH17-36	1.5 ± 1.1	0.0000	+
D27RMH17-37	9.7 ± 4.7	0.0012	+
D27RMH17-38	10.5 ± 5.5	0.0191	+
D27RMH17-39	17.5 ± 4.7	0.0042	+
D27RMH17-40	8.0 ± 4.7	0.0000	+
Minghui 63 (wide type)	30.6 ± 4.0		

[a]For most of the plants, four to five uppermost fully expanded leaves of each plant were inoculated. The lesion area was recoded at 21 d after bacterial inoculation.

[b]Each *P* value was calculated in comparison with wild type.

[c]Transgenic plants carrying the RNAi construct were detected by PCR amplification using vector-specific primers, pMCGf (5′-GGCTCACCA AACCTTAAACAA-3′) and pMCGr (5′-CTGAGCTACACATGCTCAGGTT-3′).
