# Supplementary material for: A Rice Gene of De Novo Origin Negatively Regulates Pathogen-Induced Defense Response
Source: PLoS One. 2009 Feb 25;4(2):e4603. doi: 10.1371/journal.pone.0004603 (PMC2643483; doi:10.1371/journal.pone.0004603)
Supplement: Table S3 — Sequence identity (%) among OsDR10 gene and its homologs from different species (0.09 MB PDF) [file pone.0004603.s009.pdf]

**Table S3.** Sequence identity (%) among *OsDR10* gene and its homologs from different species

| | *OsDR10* | 9311A | NipponbareA | O. rufipogonA | O. rufipogonB | 9311B | NipponbareB | Nackdong | O. punctata | O. latifolia | O. australiensis | L. tisserantii |
|---|---|---|---|---|---|---|---|---|---|---|---|---|
| 9311A | 100 | | | | | | | | | | | |
| NipponbareA | 100 | 100 | | | | | | | | | | |
| O. rufipogonA | 98 | 98 | 98 | | | | | | | | | |
| O. rufipogonB | 98 | 98 | 98 | 100 | | | | | | | | |
| 9311B | 93 | 93 | 93 | 92 | 92 | | | | | | | |
| NipponbareB | 93 | 93 | 93 | 92 | 92 | 100 | | | | | | |
| Nackdong | 93 | 93 | 93 | 92 | 92 | 100 | 100 | | | | | |
| O. punctata | 69 | 69 | 69 | 69 | 69 | 67 | 67 | 67 | | | | |
| O. latifolia | 69 | 69 | 69 | 70 | 70 | 69 | 69 | 69 | 74 | | | |
| O. australiensis | 76 | 76 | 76 | 75 | 75 | 78 | 78 | 78 | 75 | 83 | | |
| L. tisserantii | 69 | 69 | 69 | 70 | 70 | 72 | 72 | 72 | 71 | 70 | 73 | |
| L. JX | 73 | 73 | 73 | 73 | 73 | 74 | 74 | 74 | 73 | 80 | 82 | 81 |
