# Supplementary material for: A Rice Gene of De Novo Origin Negatively Regulates Pathogen-Induced Defense Response
Source: PLoS One. 2009 Feb 25;4(2):e4603. doi: 10.1371/journal.pone.0004603 (PMC2643483; doi:10.1371/journal.pone.0004603)
Supplement: Table S4 — Sequence identity/similarity (%) among OsDR10 protein and its homologs in different species (0.08 MB PDF) [file pone.0004603.s010.pdf]

Table S4. Sequence identity/similarity (%) among OsDR10 protein and its homologs in different species

	OsDR10	9311A	NipponbareA	O. rufipogonA	O. rufipogonB	9311B	NipponbareB	Nackdong	O. punctata	O. latifolia	O. australiensis	L. tisserantii
9311A	100/100											
NipponbareA	100/100	100/100										
O. rufipogonA	99/99	99/99	99/99									
O. rufipogonB	99/99	99/99	99/99	100/100								
9311B	91/91	91/91	91/91	90/90	90/90							
NipponbareB	91/91	91/91	91/91	90/90	90/90	100/100						
Nackdong	91/91	91/91	91/91	90/90	90/90	100/100	100/100					
O. punctata	66/69	66/69	66/69	65/68	65/68	63/65	63/65	63/65				
O. latifolia	61/66	61/66	61/66	61/66	61/66	59/65	59/65	59/65	67/71			
O. australiensis	68/74	68/74	68/74	67/73	67/73	68/75	68/75	68/75	68/71	82/83		
L. tisserantii	58/59	58/59	58/59	58/59	58/59	55/57	55/57	55/57	65/68	64/67	67/68	
L.JX	66/69	66/69	66/69	65/69	65/69	61/67	61/67	61/67	65/71	78/82	75/79	72/75
