# Supplementary material for: A Rice Gene of De Novo Origin Negatively Regulates Pathogen-Induced Defense Response
Source: PLoS One. 2009 Feb 25;4(2):e4603. doi: 10.1371/journal.pone.0004603 (PMC2643483; doi:10.1371/journal.pone.0004603)
Supplement: Table S5 — Gene-specific primers for qRT-PCR (0.09 MB PDF) [file pone.0004603.s011.pdf]

**Table S5.** Gene-specific primers for qRT-PCR

| Genes | Accession number | Forward primer (5'-3') | Reverse primer (5'-3') |
|---|---|---|---|
| *OsDR10* | CX109127 | TCATCAAGCTGA TTTCATCAGACA | CGTACTTGTAGAACGCCATGGA |
| *AOS2* | AY062258 | CAATACGTGTACTGGTCGAATGG | AAGGTGTCGTACCGGAGGAA |
| *CHS* | X89859 | CCGGCGAACTGCGTGTAC | TTCCTGATCTGCGACTTGTCA |
| *Cht1* | D16221 | CGTGGTGACCAACATCATCA | GAGTTGAAAGGCCTCTGGTTGT |
| *ICS1* | AK120689 | TATGGTGCTATCCGCTTCGAT | CGAGAACCGAGCTCTCTTCAA |
| *LOX* | D14000 | GCATCCCCAACAGCACATC | AATAAAGATTTGGGAGTGACATATTGG |
| *NH1* | AY923983 | CACGCCTAAGCCTCGGATTA | TCAGTGAGCAGCATCCTGACTAG |
| *PAD4* | CX118864 | GCCAGCTCCCCTACGACTTC | CGTGTGCGGTGTAGGTTGTT |
| *PAL1* | X16099 | GGGCAACCCAGTGACCAA | CGATTGCCTCGTCGGTCTT |
| *PR10/PBZ1* | D38170 | CCCTGCCGAATACGCCTAA | CTCAAACGCCACGAGAATTTG |
| *PR1a* | AJ278436 | CGTCTTCATCACCTGCAACTACTC | CATGCATAAACACGTAGCATAGCA |
| *OsWRKY13* | EF143611 | TTTGGGAAAGCGTTGATTAGT | GCGCACACACACTCCAACTC |
| *OsMPK6* | EF174189 | TTGCTACGAGGGCTAAAATATGTG | GGAACAAATTGCTTGGCTTCA |
| *Actin* | X15865 | TGTATGCCAGTGGTCGTACCA | CCAGCAAGGTCGAGACGAA |
